# In Silico, In Vitro, and In Vivo Antitumor and Anti-Inflammatory Evaluation of a Standardized Alkaloid-Enriched Fraction Obtained from *Boehmeria caudata* Sw. Aerial Parts

**DOI:** 10.3390/molecules25174018

**Published:** 2020-09-03

**Authors:** Paula P. de Paiva, Julia H. B. Nunes, Fabiana R. Nonato, Ana L. T. G. Ruiz, Rafael R. T. Zafred, Ilza M. O. Sousa, Márcia Y. Okubo, Daniel F. Kawano, Paula A. Monteiro, Mary A. Foglio, João E. Carvalho

**Affiliations:** 1 Institute of Biology, University of Campinas-UNICAMP, Campinas-SP 13083-862, Brazil; rafaelrosolen@hotmail.com (R.R.T.Z.); pa.monteiro85@gmail.com (P.A.M.); carvalho@fcf.unicamp.br (J.E.C.); 2 Chemical, Biological and Agricultural Pluridisciplinary Research Center (CPQBA), University of Campinas-UNICAMP, Paulínia-SP 13148-218, Brazil; juliahbn@gmail.com (J.H.B.N.); frnonato@uol.com.br (F.R.N.); ana.ruiz@fcf.unicamp.br (A.L.T.G.R.); ilzamo.sousa@gmail.com (I.M.O.S.); yumiokuboh@gmail.com (M.Y.O.); 3 Faculty of Pharmaceutical Sciences, University of Campinas-UNICAMP, Campinas-SP 13083-871, Brazil; dkawano@unicamp.br (D.F.K.); maryann.foglio@fcf.unicamp.br (M.A.F.); 4 Institute of Chemistry, University of Campinas-UNICAMP, Campinas-SP 13083-970, Brazil; 5 Piracicaba Dental School, University of Campinas, UNICAMP, Piracicaba-SP 13414-903, Brazil

**Keywords:** molecular docking, edema, phenanthroquinolizidine, Urticaceae, NCI-H460 cell line, myeloperoxidase

## Abstract

In the context of the cancer-inflammation relationship and the use of natural products as potential antitumor and anti-inflammatory agents, the alkaloid-enriched fraction of *Boehmeria* *caudata* (BcAEF) aerial parts was evaluated. In vitro antiproliferative studies with human tumor cell lines showed high activity at low concentrations. Further investigation on NCI-H460 cells showed an irreversible effect on cell proliferation, with cell cycle arrest at G2/M phase and programmed cell death induction. Molecular docking studies of four alkaloids identified in BcAEF with colchicine’s binding site on β-tubulin were performed, suggesting (−)-C (15*R*)-hydroxycryptopleurine as the main inductor of the observed mitotic death. In vivo studies showed that BcAEF was able to reduce Ehrlich tumor volume progression by 30 to 40%. Checking myeloperoxidase activity, BcAEF reduced neutrophils migration towards the tumor. The in vivo anti-inflammatory activity was evaluated by chemically induced edema models. In croton oil-induced ear edema and carrageenan (CG)-induced paw edema models, BcAEF reduced edema around 70 to 80% together with inhibition of activation and/or migration of neutrophils to the inflammatory area. All together the results presented herein show BcAEF as a potent antitumor agent combining antiproliferative and anti-inflammatory properties, which could be further explored in (pre)clinical studies.

## 1. Introduction

Cancer is the second leading cause of death worldwide [1], and the demand for new drugs continues to be a very important research field to pursue. In this context, plant-derived natural products play an important role, because in general they also present anti-inflammatory properties which are interesting in the context of cancer [2,3]. Inflammation is considered one of the hallmarks of this disease, which contributes to its development and progression [4]. Therefore, natural products combining antiproliferative and anti-inflammatory properties become noteworthy in the search for new anticancer agents [5,6].

Previously, the in vitro and in vivo activities of the ethanolic extract of *Boehmeria caudata* aerial parts (BcEE) were reported [7]. The extract BcEE presented in vitro antiproliferative activity against four human cell lines (one non-tumor and three tumor). Furthermore, when orally administered, BcEE reduced Ehrlich solid tumor growth with an anti-inflammatory effect, which was evaluated by the carrageenan (CG)-induced paw edema, and croton oil-induced ear edema on mice [7]. These results suggested that the antitumor activity of the BcEE could be related to the antiproliferative and anti-inflammatory effects, which may have a straight relationship with the phenanthroquinolizidine alkaloids detected in BcEE. In fact, the alkaloid-enriched fraction of *B. caudata* (BcAEF) was evaluated in vitro showing an enhanced potency when compared to BcEE [7]. Continuing the study on the pharmacological profile of *B. caudata* aerial parts, herein the in silico tubulin interaction (molecular docking studies), in vitro antiproliferative activity (2D model and flow cytometry evaluation), and in vivo antitumor and anti-inflammatory activities of BcAEF are reported for the first time, in order to attest whether the high potency observed in vitro is also maintained in vivo.

## 2. Results

### 2.1. Antiproliferative Assays

Table 1 shows the total growth inhibition (TGI) values of BcAEF (tested range: 0.0025 to 2.5 µg mL^−1^) against a panel of human tumor and non-tumor cell lines. Doxorubicin (doxo) was used as positive control. The antiproliferative profiles are in Figure S1 (Supplementary Material). BcAEF best inhibition outcome was observed on NCI-H460 (lung) and K562 (leukemia) human tumor lines.

**Figure 1:**
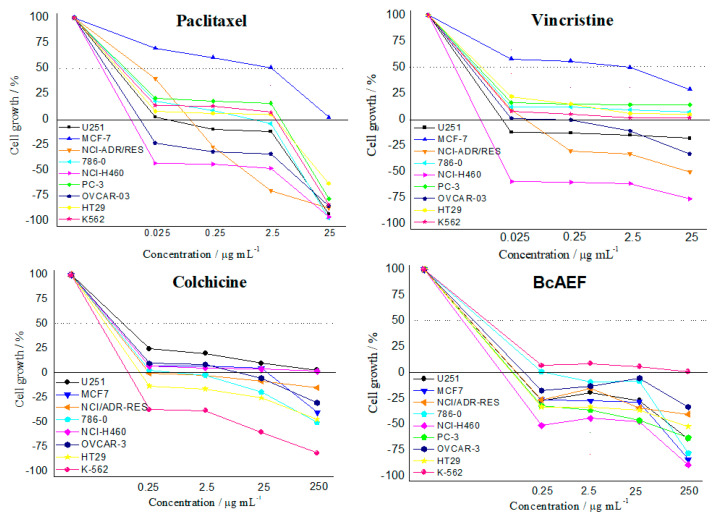
Antiproliferative profiles of paclitaxel (0.025 to 25 µg mL^−1^), vincristine (0.025 to 25 µg mL^−1^), colchicine (0.25 to 250 µg mL^−1^), and BcAEF (0.25 to 250 µg mL^−1^) after 48h-exposition over a panel of tumor cell lines. Human tumor cell lines: U251 (glioblatoma); MCF-7 (breast, adenocarcinoma); NCI-ADR/RES (multidrug resistant ovarian adenocarcinoma); 786-0 (kidney, adenocarcinoma); NCI-H460 (lung, large cells carcinoma); PC-3 (prostate, adenocarcinoma); OVCAR-3 (ovarian, adenocarcinoma); HT29 (colon, adenocarcinoma); K562 (chronic myeloid leukemia). For colchicine the PC-3 cell line is not presented due to an experimental error.

BcAEF was also tested in a higher concentration range (0.25 to 250 µg mL^−1^) together with paclitaxel (0.025 to 25 µg mL^−1^), vincristine (0.025 to 25 µg mL^−1^), and colchicine (0.25 to 250 µg mL^−1^) to observe and compare their antiproliferative profiles; the profiles were compared and resemble one another (Figure 1).

### 2.2. Cell Cycle Arrest and Clonogenic Cell Survival Assay

**Figure 2:**
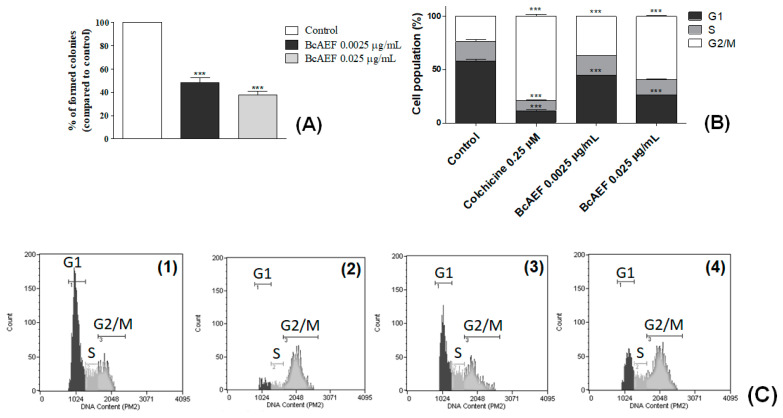
Inhibitory effects of BcAEF on NCI-H460 cell proliferation and cell cycle. (**A**) Percentage of colonies in the colony formation assay of untreated cells (control) and cells treated with 0.0025 and 0.025 µg mL^−1^ of BcAEF. (**B**) Influence on NCI-H460 cell cycle. NCI-H460 cells were treated for 30 h with 0.25 µM of colchicine (positive control), 0.0025 and 0.025 µg mL^−1^ of BcAEF. Statistical analysis: two-way ANOVA followed by Bonferroni test (*** *p* < 0.001, relative to untreated cells-control). (**C**) Histograms of cell cycle analysis (cell count vs. DNA content, Guava Cell Cycle module) of (**1**) untreated cells—control, (**2**) 0.25 µM of colchicine, (**3**) 0.0025 µg mL^−1^ and (**4**) 0.025 µg mL^−1^ of BcAEF over NCI-H460 cells (30 h), showing cell population in G1, S and G2/M phases.

Colchicine (positive control) induced cell cycle arrest at G2/M phase on NCI-H460 cells (G2/M = 24% for control, 79% for colchicine, 30 h-exposure; Figure 2B,C). BcAEF at 0.025 µg mL^−1^ induced significant cell cycle arrest at G2/M phase (G2/M = 34% and 59% after 24 and 30 h-exposure, respectively) with concomitant reduction on G1 cell subpopulation (G1 = 50% and 26% after 24 and 30 h-exposure, respectively) in a time dependent way (Figure 2B,C). At a lower concentration (0.0025 µg mL^−1^) BcAEF did not alter NCI-H460 cell cycle after 24 h-exposure (Figure S2, Supplementary Material). However, increasing time exposure to 30 h, BcAEF at 0.0025 µg mL^−1^ promoted cell cycle arrest at G2/M (G2/M from 24% for control to 37% for BcAEF) (Figure 2B,C).

Considering the clonogenic survival assay, at both concentrations BcAEF reduced in 52% and 62% (0.0025 and 0.025 µg mL^−1^, respectively) the colony formation at the 5th day (after cell exposure) in comparison to the untreated cells (Figure 2A and Figure S3, Supplementary Material).

### 2.3. Molecular Docking

**Figure 3:**
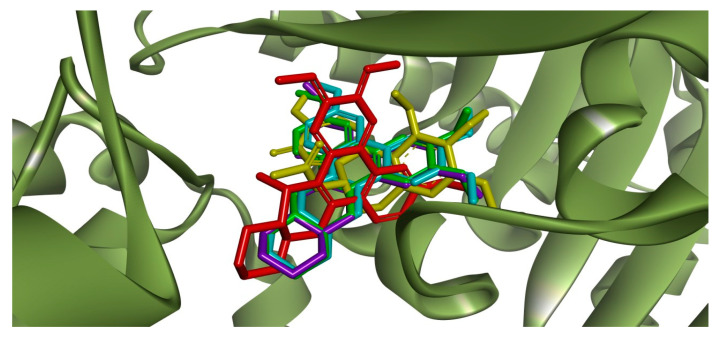
Superposition between the crystallographic pose of colchicine (yellow) at the corresponding binding site in the *β*-tubulin monomer (PDB ID: 4O2B) [8] and the docking poses of the alkaloids *R*-boehmeriasin A (green), *R*-boehmeriasin B (purple), *R*-cryptopleurine (light blue) and (−)-C (15*R*)-hydroxycryptopleurine (red).

Redocking of colchicine into the corresponding ligand site of *β*-tubulin using the GoldScore function resulted in a root mean square deviation (RMSD) = 0.44 Å, validating the docking parameters for the subsequent runs with the selected alkaloids. By analyzing the docking scores (Table S1), the affinity score was colchicine >> *R*-boehmeriasin A > (−)-C (15*R*)-hydroxycryptopleurine = *R*-boehmeriasin B > *R*-cryptopleurine. Superposition of colchicine and the other alkaloids is illustrated in Figure 3. The main intermolecular interactions performed by the selected alkaloids and colchicine with the amino acid residues from the colchicine biding site of *β*-tubulin are illustrated in Figures S4–S8 (Supplementary Material).

### 2.4. Phosphatidylserine Externalization and Caspases Activation

**Figure 4:**
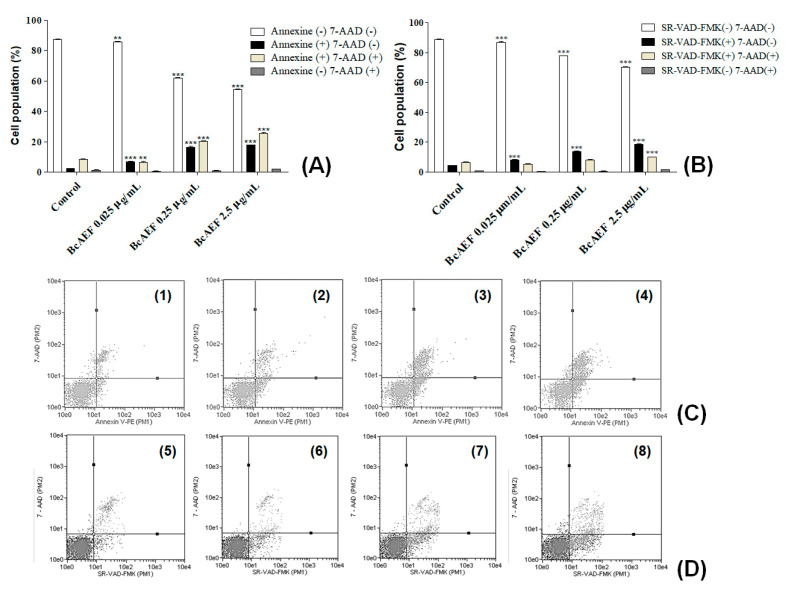
Programmed cell death effects of BcAEF on NCI-H460 cells. (**A**) Influence of BcAEF on phosphatidylserine externalization of NCI-H460 after 36 h of treatment. NCI-H460 cells were treated for 36 h with 0.025, 0.25 and 2.5 µg mL^−1^ of BcAEF. (**B**) Influence of BcAEF on caspases activation of NCI-H460 after 48 h of treatment. NCI-H460 cells were treated for 48 h with 0.025, 0.25 and 2.5 µg mL^−1^ of BcAEF. Statistical analysis for PS externalization and caspases activation: two-way ANOVA followed by Bonferroni test (** *p* < 0.01 and *** *p* < 0.001, relative to untreated cells - control). (**C**) Dot-plot graphics of cell distribution after using Annexin-V and 7-AAD dyes over NCI-H460 cells treated for 36 h (Guava Nexin Module): (**1**) untreated cells (control), (**2**) 0.025 µg mL^−1^, (**3**) 0.25 µg mL^−1^ and (**4**) 2.5 µg mL^−1^ of BcAEF. (**D**) Dot-plot graphics of cell distribution after using SR-VAD-FMK and 7-AAD dyes over NCI-H460 cells treated for 48 h (Guava MultiCaspase module): (**5**) untreated cells (control), (**6**) 0.025 µg mL^−1^, (**7**) 0.25 µg mL^−1^ and (**8**) 2.5 µg mL^−1^ of BcAEF.

After 12-h treatment (Figure S9, Supplementary Material), no PS externalization was induced by BcAEF even at the highest tested concentration (2.5 µg mL^−1^). Increasing time exposure to 24 and 36 h, a time and concentration-dependent PS exposure was observed on BcAEF-treated NCI-H460 cells (at 24 h without loss of plasmatic membrane integrity, and at 36 h with loss of plasmatic membrane integrity; Table S2, Figure S10 and Figure 4A,C). Furthermore, after 36 h and 48 h exposure BcAEF induced caspases activation without loss of plasmatic membrane integrity on NCI-H460 cells, independently on time exposure (Table S3, Figure S11 and Figure 4B,D).

### 2.5. Acute Toxicity

Following the acute toxicity protocol (OECD 425), with few adaptations, intraperitoneal administration (i.p.) of BcAEF at 300 mg kg^−1^ dose promoted convulsion and death by respiratory arrest in mice shortly after administration. These toxic effects were less intense without death occurrence when BcAEF was administrated (i.p.) at 150 mg kg^−1^ dose in mice. Moreover, all surviving mice recovered to normal behavior after 24 h in comparison to vehicle-treated animals. Changing administration route from intraperitoneal to oral (o.a.), BcAEF at 300 mg kg^−1^ dose promoted tremor and diarrhea up to 24 h after administration, progressing to death.

### 2.6. Ehrlich Solid Tumor, Myeloperoxidase Activity, and Carrageenan-Induced Paw Edema Model

**Figure 5:**
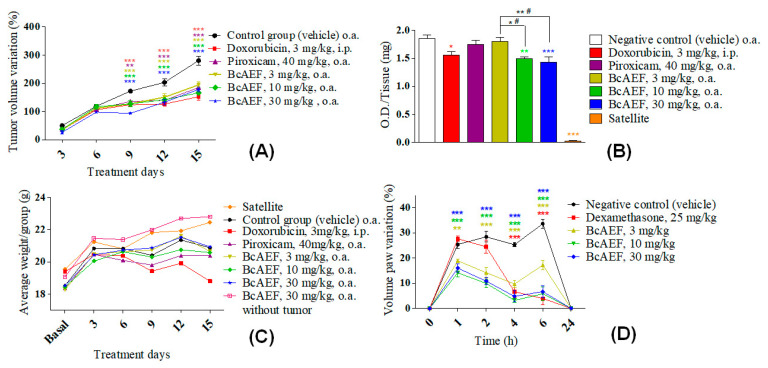
Antitumor and anti-inflammatory effects of BcAEF on Ehrlich solid tumor model and CG-induced paw edema model. (**A**) Mean tumor volume variation (%) in the Ehrlich solid tumor paw model during 15 days of treatment with vehicle (negative control), positive controls, and BcAEF. (**B**) Myeloperoxidase (MPO) activity expressed as O.D (optical density) per milligram of paw tissue with (vehicle group, positive controls and BcAEF) or without (satellite group) Ehrlich’s solid tumor. (**C**) Variation of mice body weight during 15 days of experiment of Ehrlich’s solid tumor in the paw. (**D**) CG-induced paw edema represented by edema variation (%). Results were expressed as mean ± standard error*. Groups =* vehicle (negative control), doxorubicin (positive control), piroxicam (positive control), BcAEF (3, 10, and 30 mg/kg), satellite (without inoculation of tumor cells and without treatment) and a group treated with the higher dose of BcAEF (30 mg/kg), in which tumor cells were not inoculated. Legend: i.p. intraperitoneal; o.a. oral administration. Statistical analysis: * *p <* 0.05, ** *p <* 0.01 and *** *p <* 0.001, statistically significant difference in relation to the vehicle group (two-way ANOVA followed by Bonferroni test).

By evaluating mean tumor volume variation (MTVV, %) during fifteen experimental days, all treatments (BcAEF, doxorubicin and piroxicam) promoted significant reduction in tumor growth in comparison to vehicle-treated animals from the 9th day on (Figure 5A). Considering the last experimental day (15th day), doxorubicin (chemotherapeutic agent control) inhibited tumor progression by 45.67% (152.62 ± 31.84) while piroxicam (anti-inflammatory control) reduced tumor progression by 34.13% (185.03 ± 7.56) in comparison to Erhlich-bearing mice in the vehicle group (280.91 ± 38.27). Moreover, BcAEF inhibited dose-independently the tumor progression by 30.82% (194.34 ± 24.59), 40.71% (166.56 ± 9, 85), and 36.41% (178.64 ± 21.91) at 3, 10, and 30 mg kg^−1^ respectively, in comparison to Erhlich-bearing mice in the vehicle group (280.91 ± 38.27). During the whole experiment, the animals from the experimental groups (3, 10, and 30 mg kg^−1^ doses of BcAEF and 30 mg kg^−1^ dose of BcAEF without tumor) and those treated with piroxicam showed a body weight evolution similar to that observed for naïve (Satellite group) and Ehrlich-bearing mice from the vehicle group. Only doxorubicin-treated mice showed significant loss of body weight from the 9th day of treatment, reaching 3.09% of body loss on the 15th day (Figure 5C). It is noteworthy that BcAEF at 30 mg kg^−1^ dose did not affect body weight evolution even to mice without Ehrlich tumor.

As formerly described [9], Ehrlich carcinoma cells growth promoted a large migration and infiltration of neutrophils into the tumor microenvironment, since the Ehrlich-bearing mice in the vehicle group showed approximately 60 folds more MPO activity when compared to naïve mice in the satellite group (Figure 5B). Regarding positive controls, doxorubicin-treated animals showed 16.03% of reduction in MPO activity, whereas piroxicam-treated mice did not present a significant reduction in comparison to the vehicle group (Figure 5B). Considering oral treatment with BcAEF, at 10 and 30 mg kg^−1^ doses, treated animals showed a significant reduction on MPO activity (19.35%, *p <* 0.05, and 22.90%, *p <* 0.01, respectively, Figure 5B). In a dose-independent profile, oral administration (o.a.) of BcAEF resulted in anti-edematogenic activity in the CG-induced paw edema model, corroborating the result observed in the Ehrlich solid tumor model (Figure 5D and Table S4, Supplementary Material).

### 2.7. Croton Oil-Induced Ear Edema

**Figure 6:**
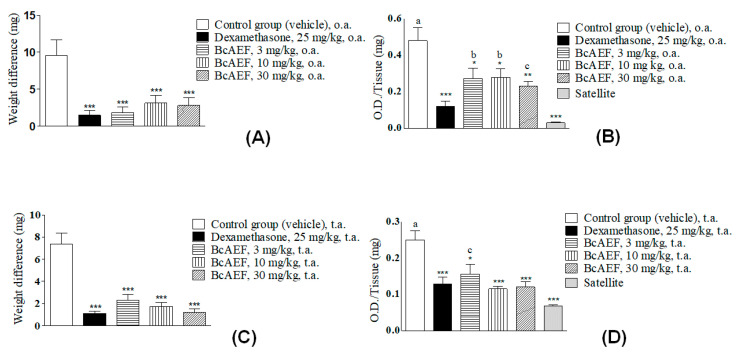
Anti-inflammatory effect of BcAEF evaluated on croton oil-induced ear edema. (**A**,**C**) Results are expressed as the mean ± standard error media; weight difference (mg) = weight differences of equal portions obtained from the treated and untreated ears of the animals from each experimental group. (**B**,**D**) Tissue sample evaluated: ear fragment after exposure to croton oil; myeloperoxidase activity expressed as O.D. (optical density) per ear milligram. Groups: negative control (vehicle = acetone 70% to group with application topic or 10 mL kg^−1^ of the PBS pH 7 + tween 80 5% to group with oral treatment), positive control (dexamethasone), and experimental group (BcAEF: alkaloid enriched fraction extracted from the aerial parts of *Boehmeria caudata Sw*). Legend: t.a. topic application; o.a. oral administration. Statistical analysis: * *p <* 0.05, ** *p <* 0.01 and *** *p <* 0.001, significant difference by statistical means according to vehicle group. a: *p <* 0.001, b: *p <* 0.01, c: *p <* 0.05, significant difference by statistical means according to satellite group (one-way ANOVA followed by Tukey test).

Regardless of the administration route (oral [o.a.] or topic [t.a.]) and doses, BcAEF reduced approximately 80% of the ear edema similarly to the reduction observed for dexamethasone (dexa) in comparison to vehicle-treated animals (Figure 6A,C). Corroborating the edema reduction, MPO activity was measured in each ear fragment collected at the end of the experiment. Dexamethasone reduced MPO activity returning to similar levels of naïve animals (Figure 6A,B) both by oral and topic routes. Furthermore, BcAEF restored MPO activity level to that observed in naïve animals when topically administrated at 10, and 30 mg mL^−1^. Oral administration of BcAEF promoted partial reduction on MPO activity level in comparison to vehicle and satellite groups (Figure 6C,D).

## 3. Discussion

Herein the antitumor and anti-inflammatory effects of standardized alkaloid-enriched fraction BcAEF prepared from the ethanolic extract of *Bohemeria caudata* aerial parts are described. *Bohemeria caudata* leaves were dried and milled, and an extraction with ethanol was done, resulting in the ethanolic extract. As formerly reported [7], *B. caudata* aerial parts ethanolic extract presented in vitro and in vivo antitumor properties, with suggestive relationship to the alkaloid’s content. Using acid-base fractionation and liquid-liquid partition with different solvents, BcAEF was produced. By high-resolution electrospray ionization mass spectrometry (HREIMS) and gas chromatography coupled to mass spectrometry (GC-MS), five phenanthroquinolizidine alkaloids (cryptopleurine, boehmeriasin A, boehmeriasin B, julandine, (−)-C(15*R*)-hydroxycryptopleurine), one quinolizidine alkaloid [3-(4-hydroxyphenyl)-4-(3-methoxy-4-hydroxyphenyl)-3,4-dehydroquinolizidine], and two acetophenone alkaloids (3,4-dimethoxy-ω-(2′-piperidyl)acetophenone and 2′,4′-dimethoxyacetophenone) were detected in the standardized fraction [7]. For the in vitro antiproliferative evaluation, the NCI-60 protocol [10] (with some adaptations) was adopted, expressing the sample activity as the concentration required to elicit total cell growth inhibition (TGI). BcAEF showed, in a first evaluation [7], a potent growth inhibitor profile against U251 (glioblastoma, TGI < 0.25 µg mL^−1^), MCF-7 (breast, TGI < 0.25 µg mL^−1^) and NCI-H460 (lung, TGI = 0.66 µg mL^−1^) tumor cell lines, similarly to that observed for the ethanolic extract. Herein, using a lower concentration range (0.0025; 0.025; 0.25 and 2.5 μg mL^−1^) and other human tumor cell lines (Table 1 and Figure S1, Supplementary Material), BcAEF presented a potent in vitro antiproliferative activity with very low TGI values, although with cytotoxicity against the non-tumor line (HaCat) as well. BcAEF was more active for NCI-H460 (large cell carcinoma of lung) and K562 (chronic myeloid leukemia) human tumor lines, with low TGI values of 0.17 and 0.11 µg mL^−1^, respectively.

Based on the NCI database of anticancer molecules, similarities in the antiproliferative profile against a tumor cell panel was postulated as an indicative of the same mechanism of action for different samples [11,12,13]. Following this hypothesis, BcAEF’s antiproliferative profile was compared with those observed for paclitaxel, vincristine, and colchicine (Figure 1). These alkaloids are known microtubule-binding agents that induce alterations in mitosis events [14]. Considering the similarities observed among the cell growth-concentration curves (Figure 1), BcAEF might be affecting mitosis process by binding microtubules.

According to Global Cancer Observatory, in 2018 lung cancer was the most frequent cancer, with 2.1 million (11.6%) new cancer cases worldwide, accounting for 1.8 million (18.4%) of all cancer deaths [1]. Considering BcAEF’s antiproliferative effect against NCI-H460 cells (large cell carcinoma of lung), this cell line was chosen for further investigation of the cell death mechanisms induced by the BcAEF.

Initially, BcAEF’s interference in the cell cycle of NCI-H460 cells after 24 and 30 h-exposure was evaluated. Colchicine, a well know compound that inhibits microtubule polymerization and assembly of the mitotic spindle [15], was used as a positive control (30 h experiment; 0.25 µM). The results demonstrated that the G2/M arresting induced by BcAEF in NCI-H460 cells was dependent on concentration and time exposure. Thereafter, using the clonogenic cell survival assay, the irreversible antiproliferative effect induced by BcAEF on NCI-H460 cells was demonstrated. These results suggested that alkaloids found in BcAEF were absorbed by NCI-H460 promoting growth inhibition even after being removed from culture medium (Figure 2).

Here, BcAEF resembled colchicine, which inhibits the polymerization of tubulin and, consequently, the process of microtubule self-assembly [15]. Considering that both BcAEF and colchicine arrested NCI-H460 cell cycle at G2/M jointly, and that phenanthroquinolizidine nuclei of BcAEF alkaloids resemble the structure of colchicine (Table S1, Supplementary Material), our hypothesis is that BcAEF alkaloids could interact with *β*-tubulin inhibiting the tubulin polymerization and, consequently, the microtubule self-assembly process. In this way, molecular docking simulations were performed for the four phenanthroquinolizidine alkaloids identified in BcAEF [*R*-cryptopleurine, (−)-C (15*R*)-hydroxycryptopleurine, *R*-boehmeriasin A, and *R*-boehmeriasin B] to verify their complementarities with the colchicine biding site in *β*-tubulin, as the last being a proposed target. Score values for colchicine and the selected alkaloids (Table S1, Supplementary Material) could provide a general outlook concerning the interactions between these compounds and the amino acid residues from the colchicine biding site of *β*-tubulin. Therefore, they could not be used to predict, quantitatively, the expected binding affinities, that would be possible only if these substances were experimentally assessed against tubulin.

The superposition of the crystallographic pose of colchicine with the most relevant docking poses for the four alkaloids (Figure 3) suggested that *R*-boehmeriasin A, *R*-boehmeriasin B and *R*-cryptopleurine adopted a very similar orientation relative to colchicine, with an almost perfect superposition between the aromatic rings of these compounds with the aromatic nucleus of colchicine. Only (−)-C (15*R*)-hydroxycryptopleurine displayed a divergent orientation. After the definition of the most representative docking pose for each alkaloid, the main intermolecular interactions performed by these compounds and colchicine with the amino acid residues from the colchicine biding site of the *β*-tubulin were studied (Figures S4–S8 for colchicine and the other alkaloids, Supplementary Material).

Visual inspection of the interactions performed by colchicine (Figure S4) and the protein target suggests the existence of a single, stronger, hydrogen bond (carbonyl oxygen with Val^181^), reinforced by several (weaker) hydrophibic interactions, which stabilize the geometry of the H-bond and, accordingly, provide an exponential increase in the affinity for the target [16]. Similar interactions were predicted in the docking simulations for *R*-boehmeriasin A (Figure S5), *R*-boehmeriasin B (Figure S6) and *R*-cryptopleurine (Figure S7), which perform more hydrophobic/aromatic interactions than the observed for colchicine. However, for these compounds the H-bonds are indirect (i.e., a water molecule acts as a bridge from the ligand to the amino acid residues) and, accordingly, the cooperative effect between the H-bond and the hydrophobic/aromatic interactions tend to be much less relevant. For (−)-C (15*R*)-hydroxycryptopleurine (Figure S8) the insertion of a hydroxyl group in the quinolizidinic ring, when compared to *R*-cryptopleurine (see the structures in Table S1), would result in a different orientation when compared to the other alkaloids (Figure 3), allowing the establishment of a H-bond between this group and the Thr^179^ residue. Additionally, another H-bond was predicted between the methoxy group of the dimethoxybenzene ring and the nitrogen from Thr^353^, besides of the existence of an indirect H-bond between the quinolizidinic N and Lys^254^. Therefore, aside from performing less hydrophobic/aromatic interactions with *β*-tubulin, (−)-C (15*R*)-hydroxycryptopleurine is expected to be a stronger tubulin inhibitor than the other alkaloids, since this compound performs more (stronger) H-bonds. This reinforces our hypothesis that, if the mode of action of BcAEF is, in fact, the inhibition of microtubule polymerization (as supported by observations concerning the similarities of the antiproliferative profiles in Figure 1 and cell cycle arrest at G2/M phase), (−)-C (15*R*)-hydroxycryptopleurine could be considered the most relevant phenanthroquinolizidine alkaloid in the BcAEF extract concerning the interaction with *β*-tubulin. However, further studies are still necessary to prove this hypothesis, since this is only a proposition.

Continuing the investigation of the effects of BcAEF over NCI-H460 cells, the cell cycle arresting was evaluated to verify whether this was the first event on the induction of regulated cell death mechanism. Hence, the externalization of phosphatydilserine (PS) was evaluated as an early stage of cell death, by using Annexin-V-PE dye combined with 7-AAD [17], despite caspases activation (Figure 4). The in silico (microtubule-binding at colchicine binding site) and the in vitro (cell cycle arrest at G2/M phase and irreversible antiproliferative effect, besides subsequent PS externalization and caspases activation) results might suggest that BcAEF promoted mitotic death in NCI-H460 cells. Mitotic death is one specific variant of regulated cell death, most often intrinsic apoptosis, characterized by the cell proliferation blocking with mitotic alterations that induce regulated cell death [18]. Further molecular evaluations could confirm this hypothesis.

Based on the potent in vitro antiproliferative effect, further in vivo studies to assess the efficiency and safety of BcAEF were done. The toxic effects observed for BcAEF in the acute toxicity protocol were quite similar to those described for pilocarpine, the alkaloid extracted from *Pilocarpus microphyllus* leaves [19]. Pilocarpine is a cholinergic agonist used as convulsive inductor in non-clinical epilepsy models [20] and to treat xerostomia by stimulation of saliva production [21]. To avoid toxic effects during pharmacological evaluations, a 30 mg kg^−1^ dose (10 folds lower than the toxic dose) of BcAEF was chosen to evidence the potential antitumor and anti-inflammatory effects.

Being a well-established model for tumor biology studies and development of anti-cancer agents [22,23,24], the solid Ehrlich tumor in mice was chosen for BcAEF’s in vivo antitumor evaluation. In comparison the observed antitumor effect of *Boehmeira caudata* ethanolic extract BcEE [7], the alkaloid-enriched fraction BcAEF showed a similar profile at lower doses (5 to 50-fold), demonstrating the efficacy afforded by fractionation (providing a more potent sample), and displaying the importance of phenantroquinolizidine and acetophenone alkaloids in the antitumor effect. Considering body weight evolution, many drugs available in cancer chemotherapy, as well as by radiotherapy, induce body weight loss. Therefore, reducing this toxic effect is one of the biggest challenges for the development of new drugs, other than higher therapeutic indexes with greater selectivity for tumor strains [25]. Thus, the absence of clinical signs of toxicity in BcAEF’s effective doses (and also not affecting body weight) represents a positive indicator for the continuity of studies.

Based on the cancer-inflammation relationship [4,26], the effect of BcAEF on the migration and/or activation of neutrophils was assessed by determining myeloperoxidase activity (MPO). The neutrophils infiltration has been observed in many types of tumors, which are called “neutrophils associated with tumors”. This condition is frequently seen in cancer associated with inflammation, where the persistence of genetically altered tumor cells, hypoxia, and cell death create an inflammatory environment with continuous leukocyte recruitment [27,28,29]. Measurement of MPO activity provides an indirect indication of the presence of neutrophils in the tumor microenvironment, since MPO is found in the granulocytes present in the neutrophils. Our results suggested that both anti-inflammatory and antiproliferative effects might have a relationship with the tumor growth reduction induced by BcAEF (Figure 5A,B).

Considering the tumor microenvironment as a therapeutic target and the relationship between cancer and inflammation [4,26,30], the anti-inflammatory activity of BcAEF using the CG-induced mice paw edema and croton oil-induced mice ear edema models was evaluated. Similarly to the Ehrlich solid tumor model, oral administration (o.a.) was chosen for all treatments to avoid intraperitoneal-induced local inflammatory process. Described originally in 1962 [31], the CG-induced inflammation model is acute, nonimmune, well-researched, and highly reproducible [32]. After injected, CG induces edema, hyperalgesia, and erythema by promoting pro-inflammatory agent release, such as bradykinin, histamine, and tachykinins. Moreover, neutrophils migration is stimulated resulting reactive oxygen species generation and increasing inflammatory response. Experimentally, the inflammatory response is usually quantified by paw size increase (edema), which maximal effect is obtained approximately 4 to 6 h post CG-injection. The CG-induced inflammatory cascade can be modulated by inhibition of lipoxygenases (LOX) and/or cyclooxygenases (COX), for example, being a robust reproductive model for research of both steroidal and non-steroidal (NSAIDs) anti-inflammatory drugs [32,33,34]. Once again, the alkaloid-enriched fraction BcAEF demonstrated to be a better anti-inflammatory agent (around 1.5 to 15 fold) when compared to the ethanolic fraction BcEE [7].

Furthermore, BcAEF’s anti-inflammatory activity was assessed by the croton oil-induced ear edema model in mice (Figure 6). This model allowed for the evaluation of both topical and systemic routes of steroidal and non-steroidal anti-inflammatory drugs [34,35,36]. The inflammatory reaction induced by the phorbol esters of croton oil is associated with neutrophil infiltration, alterations in cytokine production, and increased prostaglandins and leukotrienes production [37,38]. Therefore, substances with LOX, COX and/or phospholipase A2 inhibitory effects can be evaluated in this model [34,39]. Both antitumor and anti-inflammatory activities evidenced for BcAEF could be partially related to the presence of phenanthroquinolizidine alkaloids. Several alkaloids of this class have been described as potent antiproliferative, antiviral, and anti-inflammatory agents [40,41,42]. Moreover, the indirect evidence of neutrophil chemotaxis inhibition by reduction of MPO activity observed for BcAEF was similar to that reported for colchicine [43], corroborating the in silico interaction with colchicine-binding site of microtubules and the in vitro antiproliferative profile with cell cycle arrest.

## 4. Materials and Methods

### 4.1. General Experimental Procedures

All solvents and chemicals were purchased from commercial sources and were used without further purification. Dichloromethane and ethanol (analytical grade) were obtained from Labsynth (Diadema, SP, Brazil). Trichloroacetic acid, Tween 80^®^, dexamethasone (purity > 90%), dimethylsulfoxide (DMSO), carrageenan, croton oil and hexadecyltrimethylammonium bromide buffer (HTAB) were obtained from Sigma-Aldrich Laboratories (San Loius, MO, USA). Piroxicam was obtained from Pfizer (New York, NY, USA). Culture medium RPMI 1640 and fetal bovine serum (SBF) were from Gibco (Waltham, MA, USA). Penicillin/streptomycin (1000 U/mL:1000 mg/mL) and trypsin-EDTA 0.25% were acquired from Vitrocell (Campinas, SP, Brazil). Doxorubicin (chlorohydrate 50 mg; purity > 90%) was obtained from Eurofarma (São Paulo, SP, Brazil). Crystal violet, Guava Nexin Reagent, Guava Multicaspase kit (Caspase Reagent Working Solution, Apoptosis Wash Buffer and Caspase 7-AAD Reagent Working Solution) and Guava Cell Cycle Reagent were acquired from Merck-Millipore (Darmstadt, Germany). Flow cytometry experiments and analysis were performed using a Guava EasyCyte Mini Flow Cytometry System (Millipore). Plethysmometer apparatus (7140) was purchased from Ugo Basile (Gemonio, VA, Italy) and a grinder (Disperser T 10 basic) from IKA (Campinas, SP, Brasil).

### 4.2. Plant Material

The aerial parts of *B. caudata Sw.* were collected from the CPQBA-UNICAMP experimental field (Paulínia, SP, Brazil). The botanical identification was performed by Jorge Yoshio Tamashiro, MSc., from the Department of Plant Biology, Institute of Biology, UNICAMP. A voucher specimen is deposited at the UNICAMP Herbarium under number UEC 107966. The present study was approved by the Genetic Patrimony Management Board (CGEN/MMA) through the Access and Shipment Component of Genetic Heritage for scientific research purposes (#010672/2012-5). This information is the same used in a previous publication [7].

### 4.3. Production of the Alkaloid Enriched Fraction of B. caudate and Acid-Base Extraction

Extract preparation and acid-base fractionation of *B. caudata* alkaloid-enriched fraction (BcAEF) were performed according to the methods described in our previous publication, with some modifications [7]. Milled dried aerial parts of *B. caudata* (100 g) were successively extracted with 95% ethanol using a Soxhlet extraction system (1:5 plant: solvent ratio, *w*/*v*). Solvent evaporation under low pressure at 40 °C and freeze-drying resulted in the ethanolic extract (EE, 15.70% ± 1.93% yield). The EE was diluted in distilled water (1:4, plant: solvent ratio, *w*/*v*) and acidified with 10% hydrochloric acid to pH ≈ 1. After 24 h, the mixture was vacuum filtered at 4 °C to separate a dark green precipitate (which was discarded) from the red supernatant. The latter was extracted by liquid-liquid partitioning with ethyl acetate (3:1, *v*/*v*, 3 times), affording the aqueous acidic solution (AAS), and the ethyl acetate solution (discarded). Furthermore, the AAS was partitioned with ethyl ether (3:1, *v*/*v*, 3 times) providing ethyl ether solution (discarded), and the final aqueous acidic solution (AAS_F_). After pH adjustment (pH ≈ 11) with ammonium hydroxide, AAS_F_ was partitioned with dichloromethane (3:1, *v*/*v*, 3 times) providing the aqueous basic and dichloromethane (DCM) solutions. After washing with distilled water (3:1, *v*/*v*, 3 times) and filtered through anhydrous sodium sulfate, the DCM solution was evaporated to dryness under vacuum at 40 °C. The characterization of the alkaloid enriched fraction by high-resolution electrospray ionization mass spectrometry and gas chromatography coupled to mass spectrometry is described in our previous publication [7]. After characterization the DCM fraction was renamed as alkaloid enriched fraction (AEF 0.46% ± 0.09% yield). Figure S12 shows a scheme for the extraction process. This procedure was repeated several times to obtain sufficient mass for the experiments. BcAEF was used in the in vitro and in vivo experiments herein reported.

### 4.4. Cell Culture and Sample Preparation for In Vitro Experiments

BcAEF was tested in nine human tumor cell lines: U251 (glioblastoma), MCF-7 (breast, adenocarcinoma), NCI-ADR/RES (multi-drug resistant ovarian adenocarcinoma), 786-0 (kidney, adenocarcinoma), NCI-H460 (lung, large cells carcinoma), PC-3 (prostate, adenocarcinoma), OVCAR-03 (ovarian, adenocarcinoma), HT29 (colon adenocarcinoma) and K562 (chronic myeloid leukemia)], kindly provided by Frederick Cancer Research & Development Center, National Cancer Institute, Frederick, MD, USA. The human non-tumor cell line HaCat (immortalized keratinocyte) used for antiproliferative activity assay was provided by Dr. Ricardo Della Coletta (University of Campinas-UNICAMP, Brazil). Stock cultures were grown in RPMI-1640 supplemented with 5% FBS and 1% penicillin:streptomycin (complete medium) at 37 °C in 5% CO_2_. These conditions were used both for cell line maintenance and for experiments. Cells were used in passages 4 to 10. For all in vitro experiments, aliquots (2.0 mg) of BcAEF were previously dissolved in dimethylsulfoxide (DMSO) followed by serial dilution in complete medium, affording the final concentrations for each experiment. The DMSO final concentration was always lower than 0.25% to avoid interference on cell viability. The concentrations used for colony formation assay and flow cytometry experiments were chosen based on the antiproliferative profile of BcAEF (Figure S1, Supplementary Material).

### 4.5. Antiproliferative Activity

The antiproliferative activity of BcAEF was tested on all nine human tumor cell lines and in the non-tumor line, being determined by the sulforhodamine B assay as previously described [7,44]. The TGI values (concentration that inhibits 100% cell growth) were determined through non-linear regression, type sigmoidal, analyzed using Origin 8.0 software (OriginLab Corporation, Northampton, MA, USA) [7,45]. BcAEF was tested in final concentrations of 0.0025, 0.025, 0.25 and 2.5 µg mL^−1^. Doxorubicin (final concentrations 0.025, 0.25, 2.5 and 25 µg mL^−1^) was used as a control for cell growth. In another experiment, for the comparison of the antiproliferative profiles of different alkaloids, paclitaxel (final concentrations 0.025, 0.25, 2.5 and 25 µg mL^−1^), vincristine (final concentrations 0.025, 0.25, 2.5 and 25 µg mL^−1^), colchicine (final concentrations 0.25, 2.5, 25 and 250 µg mL^−1^) and BcAEF (final concentrations 0.25, 2.5, 25 and 250 µg mL^−1^) were tested in all nine human tumor cell lines. In each experiment all compounds were tested in triplicates.

### 4.6. Colony Formation Assay

This procedure was performed according to Nunes et al. with a few adaptions [17]. The colony counting was done after 5 days of cell growth representing about 6.7 cell cycles considering the doubling time for NCI-H460 as 17.8 h [46]. NCI-H460 cells were seeded on T25 flasks (5 × 10^4^ cells/mL, 5 mL/flask) and incubated to reach ~ 80% confluence. Three T25 flasks were prepared, being one for each treatment. Cells were treated in the flasks with 0.0025 and 0.025 µg mL^−1^ of BcAEF for 24 h. Complete medium (RPMI-1640 supplemented with 5% FBS and 1% penicillin:streptomycin) with 0.25% of DMSO was used as control. After trypsinization, each resulting cell suspension was adjusted to 100 cells/well and seeded in triplicate in 6-well microplates. Cells were allowed to grow during 5 days. Finally, cells were rinsed with DPBS, fixed with methanol/DPBS 1:1 for 2 min, and afterwards with methanol for 10 min. Cells were stained with 0.1% crystal violet for 30 min and washed with water. Using a light microscope, the number of colonies was counted, considered as one grouping with at least 50 cells. Results were presented as the percentage of grown colonies compared to the control (untreated cells).

### 4.7. Cell Cycle Arrest

The procedure was adapted from Nunes et al. and Franco et al. [17,47]. NCI-H460 cells (5 × 10^4^ cells/mL) were seeded on 6-well microplates (2 mL/well) and incubated. After 24 h, cell medium was changed to RPMI-1640 supplemented with 1% penicillin:streptomycin without FBS and cells were incubated further 24 h. Thereafter, cells were treated in triplicates with 0.0025 and 0.025 µg mL^−1^ of BcAEF for 24 and 30 h. Complete medium with 0.25% DMSO was used as negative control, while 0.25 µM of colchicine was used as positive control for the 30 h experiment. After each exposition time, cells were harvested with trypsin-EDTA 0.25%, washed with PBS and fixed with ethanol 70% for at least 12 h. After washing with PBS, each treated or untreated cellular pellet was suspended in 200 µL Guava Cell Cycle Reagent, kept in the dark during 20 min at room temperature and analyzed in the flow cytometer (5000 events per replicate; fluorescence detection: 650–670 nm for PI). Gating was done according to the GuavaSoft software (Guava Cell Cycle module, Darmstadt, Germany) from EasyCyte Mini Flow Cytometry System.

### 4.8. Phosphatidylserine Externalization

The procedure was adapted from Nunes et al. [17]. NCI-H460 cells (5 × 10^4^ cells/mL) were seeded on 12-well microplates (1 mL/well) and incubated for 24 h. Thereafter, cells were treated with 0.0025, 0.025 and 2.5 µg mL^−1^ of BcAEF for 12, 24 and 36 h. Thenceforth, each exposition time cells were harvested with trypsin-EDTA 0.25%, washed with PBS and re-suspended in 100 µL Guava Nexin Reagent (Annexin-V PE/7-AAD) for 20 min at room temperature and in the dark before injection in the flow cytometer (2000 events per replicate; fluorescence detection: 525–575 nm for PE and 650–670 nm for 7-AAD). Gating was done according to the GuavaSoft software (Guava Nexin module, Darmstadt, Germany) from EasyCyte Mini Flow Cytometry System.

### 4.9. Detection of Activated Caspases

The procedure was adapted from Nunes et al. [17]. NCI-H460 cells (5 × 10^4^ cells/mL) were seeded on 12-well microplates (1 mL/well) and incubated for 24 h. Cells were then treated with 0.0025, 0.025 and 2.5 µg mL^−1^ of BcAEF for 36 and 48 h. Afterwards, cells were trypsinized, washed with PBS and re-suspended in 10 µL of Guava Caspase Reagent Working Solution. After 1h-incubation in the dark at 37 °C and 5% CO_2_, cells were washed with 100 µL 1x Apoptosis Wash Buffer and re-suspended in 200 µL Caspase/7-AAD Reagent Working Solution. After 10 min at room temperature in the dark, each cell suspension was analyzed by the flow cytometer (5000 events per replicate; fluorescence detection: 590–600 nm for SR-VAD-FMK and 650–670 nm for 7-AAD). Gating was done according to the GuavaSoft software (Guava MultiCaspase module) from EasyCyte Mini Flow Cytometry System.

### 4.10. Molecular Docking Simulations

In silico studies were performed to predict the interaction profiles for the phenanthroquinolizidine alkaloids *R*-cryptopleurine, (−)-C (15*R*)-hydroxycryptopleurine, *R*-boehmeriasin A, and *R*-boehmeriasin B with the amino acid residues from *β*-tubulin colchicine binding site. Ligand preparation and docking simulations were performed as we previously described [48], while the docking runs were based on the crystallographic monomeric structure of β-tubulin complexed with colchicine (PDB ID: 4O2B) [8], assessed in a 10 Å radius docking sphere.

### 4.11. Animals and Drugs for In Vivo Experiments

Experiments were conducted with Balb/c (17–25 g, 60 days), and Swiss (25–35 g, 60 days) female mice obtained from the Multidisciplinary Center for Biological Investigation at Animals Sciences Laboratory (CEMIB–UNICAMP). Animals were maintained at the Animal Facilities of Pharmacology and Toxicology Division, CPQBA-UNICAMP, under controlled conditions [temperature 25 ± 2 °C for 12 h light/dark cycle, with free access to food (Nuvilab^®^, Colombo, PR, Brazil) and tap water]. Animal care, research and animal sacrifice protocols were in accordance with the principles and guidelines adopted by the Brazilian College of Animal Experimentation (COBEA). The experimental procedures were approved by the Institute of Biology/UNICAMP—Ethical Committee for Animal Research (3440-1, 3441-1, 3391-1 and 3390-1). Euthanasia was performed by deeping anesthesia followed by cervical dislocation. This procedure was the same used in our previous publication [7]. BcAEF was emulsified with Tween 80 (5% final concentration) followed by dispersion in phosphate-buffered saline (PBS, pH 7.0) to afford the selected doses. The positive controls doxorubicin (chemotherapeutic agent), dexamethasone and piroxicam (anti-inflammatory agents) were prepared as described for BcAEF, while the negative control was Tween 80^®^ 5% in PBS (vehicle). For all experiments, the maximum volume administrated for animals, by oral (o.a.) or intraperitoneal (i.p.) routes, was 10 mL kg^−1^. Four-hour fasting period with free access to water was applied only for experiments with single oral administration (acute treatment), to allow total gastric emptying and no feeding interference in the active principles absorption. Moreover, all anti-inflammatory experimental models began 1 h after drug administration to ensure absorption and systemic distribution. For dose-repeated experiments, instead of the fasting period, animals were treated in the late afternoon, period in which animals presented an empty stomach.

### 4.12. Acute Toxicity Evaluation

The procedure was adapted from Paiva et al. [7]. Swiss mice were distributed (*n* = 5 animals/group) into negative control (vehicle, 10 mL kg^−1^) and experimental (BcAEF, 300 mg/kg, o.a.; 150 and 300 mg/kg doses, i.p.) groups. After administration, animals were observed during the first four hours, and daily during 14 days. On the following day, all animals were euthanized by deeping anesthesia followed cervical dislocation [OECD 425, 2014].

### 4.13. Ehrlich Solid Tumor Model

The procedure was adapted from Paiva et al. [7]. The Ehrlich tumor cells were maintained in the ascitic form by weekly intraperitoneal transplantations in *Swiss* mice. For the experiments, the ascitic suspension was collected and tumor cell suspension was prepared in PBS at a density of 2.5 × 10^6^ cells/30 µL/animal after cell viability evaluation with trypan blue. Before cell inoculation, basal volumes of the right hind paw were measured in a plethysmometer apparatus. In sequence, 2.5 × 10^6^ viable tumor cells in a volume of 30 µL were injected in the subplantar site of the right hind paw of Balb/C female mice (*n* = 8 per group). On the 3rd day after tumor inoculation, the animals were distributed randomly in four groups: vehicle (negative control, 10 mL kg^−1^, o.a.), doxorubicin (positive control, 3 mg kg^−1^, i.p.), piroxicam (positive control, 40 mg kg^−1^, o.a.), BcAEF (3, 10 and 30 mg kg^−1^, o.a.). All groups were treated every three days. The tumor volume (expressed in mL) was measured every three days using the plethysmometer apparatus until the 15th day, when the animals were sacrificed. The variation in volume was determined by the difference between the volume measured on the day and the basal volume, and then the variation in tumor volume was calculated according to the following formula [49]:

(1) $$Variation\;of\;the\;tumor\;growth\left(\%\right):\frac{Volume\;measured-basal\;volume}{basal\;volume\;}\times 100$$

On the 16th day, after the last evaluation of the animals’ paw volume, the mice were weighed and euthanized. Both hind paw were removed, weighed and stored in liquid nitrogen for the measurement of myeloperoxidase. For the tumor inhibition rate analysis, calculation was done only for the last measurement of the paw volume (15th day), according to the following formula:

(2) $$Inhibition\;of\;paw\;tumor\;\left(\%\right):\;\frac{MVTC-MVTT}{MVTC}\;\times \;100$$

where *MVTC* is the mean of tumor volume variation of the control group paw, and *MVTT* is the mean of the tumor volume variation of the treated group paw.

In order to obtain data on BcAEF’s toxicity, the body weight evolution of animals treated and inoculated with cells was compared with two groups (*n* = 8/animals), namely: satellite group (without inoculation of tumor cells and without treatment) and a group treated with the higher dose of BcAEF (30 mg kg^−1^), in which tumor cells were not inoculated. The evolution of body weight was performed by weighing all animals every three days until the end of the experiment, on a semi-analytical balance.

### 4.14. Carrageenan-Induced Paw Edema

The procedure was adapted from Paiva et al. and Lin et al. [7,50]. Balb/c female mice (*n* = 8/group) were weighed, randomly distributed and orally treated with vehicle (10 mL kg^−1^, negative control), dexamethasone (25 mg/kg, positive control) and BcAEF (3, 10 and 30 mg kg^−1^). After one hour, inflammation was induced by CG inoculation (3% in PBS, 30 µL/paw) into the sub-plantar region of the right hind footpad. The hind paw volume was evaluated using a plethysmometer at 0 (basal volume), 1, 2, 4, 6, and 24 h after CG inoculation and the edema volume was obtained by the difference between basal and experimental hind paw volumes.

### 4.15. Croton Oil-Induced Ear Edema

The procedure was adapted from Paiva et al. [7]. Balb/c mice (*n* = 6 animals/group) were weighed and distributed into the experimental groups (Table 2). The mice received croton oil topical application (20 µL/ear, 5% in acetone 70%) on the right ear one hour after each treatment, while the left ear was treated with 70% acetone (20 µL/ear). After four hours of croton oil application, the animals were euthanized and the ear edema was determined by weight difference between the left and right ears. Immediately after weighing, the ear fragments were stored in liquid nitrogen for the measurement of myeloperoxidase. A satellite group (without treatment) was also used for the evaluation of myeloperoxidase.

### 4.16. Myeloperoxidase Activity Assay—Assessment of Neutrophil Activation/Migration

The myeloperoxidase (MPO) dosage was performed using the methodology described by Bradley et al. [51]. The ear fragments (ear edema by croton oil) and paws (Ehrlich solid tumor) obtained from experimental groups treated with BcAEF were compared to the control groups and satellite group.

For the MPO quantification, frozen tissue fragments (ear or paw—100 mg) were mixed with 1 mL of reaction buffer (0.5% HTAB-hexadecyltrimethylammonium bromide buffer in 50 mM potassium phosphate buffer, pH 6.0). Samples were homogenized in sonication (IKA grinder) and then centrifuged for 10 min, 12,000 rpm at 4 °C. In a 96-well microplate, 30 μL/well of the supernatant of each sample was pipetted in triplicate and 270 μL/well substrate buffer (0.167 mg mL^−1^ ortho-dianisin and 0.002% H_2_O_2_ in phosphate buffer) were added to the preparation. For the blank, 270 μL/well of the substrate buffer plus 30 μL of the buffer with HTAB were used. The plate was incubated at 37 °C for 5 min. To stop the reaction, 50 μL of 4M sulfuric acid was used. The reading was performed at 450 nm in a spectrophotometer.

To evaluate the reduction rate of migration and/or activation of neutrophils to the inflamed site, the following formula was used:

(3) $$Reduction\;rate\;of\;the\;neutrophil\;migration\;\left(\%\right):\;\frac{MCG-MGE}{MCG}\;\times \;100$$

where *MCG* is the mean optical density obtained from the negative control; *MGE* is the mean optical density obtained from the experimental groups.

### 4.17. Statistical Analysis

All experimental results were expressed as the mean ± standard error media (SEM) or mean ± standard deviation (SD). Statistical significance was evaluated by analysis of variance (ANOVA), one-way or two-way, followed by Tukey or Bonferroni tests, using the software GraphPad Prism version 5.0 (GrapgPad Software, Inc., San Diego, CA, USA). Statistical significance was represented by * *p <* 0.05, ** *p <* 0.01 and *** *p <* 0.001.

## 5. Conclusions

BcAEF showed a significant antiproliferative activity in all human tumor lines evaluated, with the most relevant results for K-562 (chronic myeloid leukemia) and NCI-H460 (lung, large cells carcinoma) cell lines. The effect of BcAEF caused cell cycle arrest at G2/M phase on NCI-H460 cell line, granting an irreversible antiproliferative effect (as observed in colony formation assay), with further triggering of programmed cell death, shown by PS externalization and caspases activation. This provided a deeper understanding of the alkaloid enriched fraction mechanism of action. Comparison of BcAEF to NCI60 antiproliferative profile of other alkaloids demonstrated similarity to colchicine. Therefore, the prediction that compounds present in BcAEF could have the same cell target as colchicine prompted in silico studies. The docking simulations of boehmeriasin A, boehmeriasin B, and cryptopleurine (all present in BcAEF) suggested that these compounds probably act at the colchicine site, nevertheless with less inhibition potency than colchicine itself. As for (−)-C (15*R*)-hydroxycryptopleurine, although the docking score values suggested that this compound is less potent than colchicine itself, visual analysis of the interactions suggests a stronger interaction pattern when compared to the other phenantroquinolizidine alkaloids, possibly with this alkaloid being the main mitotic agent responsible for BcAEF’s activity. The in vitro antiproliferative activity observed for tumor growth was corroborated when evaluated in animal experimental solid Ehrlich tumor model, with decrease of tissue neutrophils’ infiltration, demonstrating BcAEF’s relationship of tumor inhibition with anti-inflammatory mechanisms. That supposition was endorsed by evaluation in two edema models, that demonstrated BcAEF’s anti-inflammatory activity with neutrophils migration inhibition. No clinical signs of toxicity were observed for repeated dose administration of the highest 30 mg kg^−1^ dose. The results presented herein encourage further in-depth studies on the fraction’s mechanism of action.

## 6. Patents

Patent application filed with the INPI by INOVA-UNICAMP (BR 10 2017 018240 1).

## Figures and Tables

**Table 1:** Antiproliferative activity of BcAEF and doxorubicin expressed as TGI values (µg mL^−1^).

Compounds	Cell Lines *									
	2	M	A	7	4	P	O	H	K	Cat
BcAEF	0.59	0.61	>2.5	>2.5	0.17	0.32	0.79	0.24	0.11	0.19
Doxorubicin	>25	2.83	>25	1.88	>25	0.83	1.23	>25	0.93	0.78

* Human tumor cell lines: 2 = U251 (glioblatoma); M = MCF-7 (breast, adenocarcinoma); A = NCI/ADR-RES (multidrug resistant ovarian adenocarcinoma); 7 = 786-0 (kidney adenocarcinoma); 4 = NCI-H460 (lung, large cells carcinoma); P = PC-3 (prostate, adenocarcinoma); O = OVCAR-3 (ovarian adenocarcinoma); H = HT29 (colon, adenocarcinoma); K = K562 (chronic myeloid leukemia); Non-tumor human line: Cat = HaCat (immortalized keratinocytes). TGI: concentration that inhibits 100% of cell growth after 48h-exposition. TGI values were calculated by sigmoidal regression analysis using ORIGIN 8.0 (OriginLab Corporation).

**Table 2:** Group distribution in the croton oil induced ear edema assay in mice.

Route of Administration	Group ^a^		
	Vehicle ^b^	Dexamethasone ^c^	BcAEF ^d^
Topic application	20 µL	5 mg/mL	3,10, 30 mg/mL
Oral administration	10 mL/kg	25 mg/kg	3,10, 30 mg/kg

**^a^***n* = 6 Balb/c mice/groups; **^b^** Negative control (70% acetone on topical administration and PBS with 5% Tween 80 by oral administration; **^c^** Positive control and **^d^** alkaloid enriched fraction of *B. caudata*.

## Supplementary Materials

The following are available online, Figure S1: Cell growth profiles after 48h-exposition to (A) BcAEF (0.0025 to 2.5 µg mL^−1^) and (B) doxorubicin (10^−3^ to 25 µg mL^−1^); Figure S2. Influence of BcAEF on NCI-H460 cell cycle; Figure S3. NCI-H460 colonies grown after 5 days, from (A) untreated cells (control), cells treated with (B) 0.0025 and (C) 0.025 µg mL^−1^ of BcAEF; Figure S4. Main intermolecular interactions between colchicine and the amino acid residues from the colchicine biding site of β-tubulin; Figure S5. Main intermolecular interactions between *R*-boehmeriasin A and the amino acid residues from the colchicine biding site of *β*-tubulin.; Figure S6. Main intermolecular interactions between *R*-boehmeriasin B and the amino acid residues from the colchicine biding site of *β*-tubulin; Figure S7. Main intermolecular interactions between *R*-cryptopleurine and the amino acid residues from the colchicine biding site of *β*-tubulin; Figure S8. Main intermolecular interactions between (−)-C (15*R*)-hydroxycryptopleurine and the amino acid residues from the colchicine biding site of *β*-tubulin.; Figure S9. Influence of BcAEF on on phosphatidylserine externalization of NCI-H460 after 12 h of treatment; Figure S10. Influence of BcAEF on phosphatidylserine externalization of NCI-H460 after 24 h of treatment; Figure S11. Influence of BcAEF on caspases activation of NCI-H460 after 36 h of treatment; Figure S12. Scheme for BcAEF extraction from *Boehmeria caudata* Sw. aerial parts; Table S1. Chemical structures and the respective docking scores calculated using GoldScore function (Gold 5.3 software) for colchicine, *R*-boehmeriasin A, and *R*-boehmeriasin B, *R*-cryptopleurine, and (−)-C (15*R*)-hydroxycryptopleurine; Table S2. Cell population distribution after using Annexin-V and 7-AAD dyes over NCI-H460 cells treated for 24 h and 36 h with BcAEF (0.025, 0.25 and 2.5 µg mL^−1^); Table S3. Cell population distribution after using SR-VAD-FMK and 7-AAD dyes over NCI-H460 cells treated for 36 h and 48 h with BcAEF (0.025, 0.25 and 2.5 µg mL^−1^); Table S4. Evaluation of edema variation and inhibition rate (%) promoted by BcAEF in the carrageenan-induced paw edema model; Table S5. Evaluation of reduction of edema, reduction rate of the neutrophil migration and the inhibition rate (%) promoted by BcAEF in the croton oil-induced ear edema model.

## References

[1] Bray F., Ferlay J., Soerjomataram I., Siegel R.L., Torre L.A., Jemal A. (2018). Global cancer statistics 2018: GLOBOCAN estimates of incidence and mortality worldwide for 36 cancers in 185 countries. CA Cancer J. Clin..

[2] Aravindaram K., Yang N.S. (2010). Anti-inflammatory plant natural products for cancer therapy. Planta Med..

[3] Ouyang L., Luo Y., Tian M., Zhang S.-Y., Lu R., Wang J.-H., Kasimu R., Li X. (2014). Plant natural products: From traditional compounds to new emerging drugs in cancer therapy. Cell Prolif..

[4] Hanahan D., Weinberg R.A. (2011). Hallmarks of cancer: The next generation. Cell.

[5] De Lima R.M.T., Dos Reis A.C., De Menezes A.-A.P.M., Santos J.V.D.O., Filho J.W.G.D.O., Ferreira J.R.D.O., De Alencar M.V.O.B., Da Mata A.M.O.F., Khan I.N., Islam A. (2018). Protective and therapeutic potential of ginger (*Zingiber officinale*) extract and [6]-gingerol in cancer: A comprehensive review. Phyther. Res..

[6] De Oliveira Aragão D.M., De Assis Lima I.V., Da Silva J.M., Bellozi P.M.Q., De Carvalho da Costa J., Cardoso G.M.M., De Souza-Fagundes E.M., Scio E. (2013). Anti-inflammatory, antinociceptive and cytotoxic effects of the methanol extract of *Cecropia pachystachya* Trécul. Phyther. Res..

[7] De Paiva P.P., Nonato F.R., Ruiz A.L.T.G., De Oliveira Sousa I.M., Zafred R.R.T., De Oliveira D.N., Catharino R.R., Foglio M.A., De Carvalho J.E. (2020). An ethanolic extract of *Boehmeria caudata* aerial parts displays anti-inflammatory and anti-tumor activities. Planta Medica Int. Open.

[8] Prota A.E., Franck D., Bachmann F., Bargsten K., Buey R.M., Pohlmann J., Reinelt S., Lane H., Steinmetz M.O. PDB Entry—4O2B. https://www.wwpdb.org/pdb?id=pdb_00004o2b.

[9] Mal’tseva V.N., Avkhacheva N.V., Santalov B.F., Safronova V.G. (2006). Dynamic analysis of modification of peripheral neutrophils functional activity and its regulation during tumor growth in vivo. Tsitologiia.

[10] National Cancer Institute NCI60 Methodology. https://dtp.cancer.gov/discovery_development/nci-60/methodology.htm.

[11] Paull K.D., Shoemaker R.H., Hodes L., Monks A., Scudiero D.A., Rubinstein L., Plowman J., Boyd M.R. (1989). Display and analysis of patterns of differential activity of drugs against human tumor cell lines: Development of mean graph and COMPARE algorithm. J. Natl. Cancer Inst..

[12] Zaharevitz D.W., Holbeck S.L., Bowerman C., Svetlik P.A. (2002). COMPARE: A web accessible tool for investigating mechanisms of cell growth inhibition. J. Mol. Graph. Model..

[13] Huang R., Wallqvist A., Covell D.G. (2006). Assessment of in vitro and in vivo activities in the national cancer institute’s anticancer screen with respect to chemical structure, Target Specificity, and mechanism of action. J. Med. Chem..

[14] Yue Q.X., Liu X., Guo D.A. (2010). Microtubule-binding natural products for cancer therapy. Planta Med..

[15] Molad Y. (2002). Update on colchicine and its mechanism of action. Curr. Rheumatol. Rep..

[16] Muley L., Baum B., Smolinski M., Freindorf M., Heine A., Klebe G., Hangauer D.G. (2010). Enhancement of hydrophobic interactions and hydrogen bond strength by cooperativity: Synthesis, modeling, and molecular dynamics simulations of a congeneric series of thrombin inhibitors. J. Med. Chem..

[17] Bormio Nunes J.H., De Paiva P.P., Ruiz A.L.T.G., De Carvalho J.E., Corbi P.P. (2019). New findings on the antiproliferative activity of the silver(I) complex with 5-fluorouracil against human multi-resistant NCI/ADR-RES ovarian tumor cells. Toxicol. Vitr..

[18] Galluzzi L., Vitale I., Aaronson S.A., Abrams J.M., Adam D., Agostinis P., Alnemri E.S., Altucci L., Amelio I., Andrews D.W. (2018). Molecular mechanisms of cell death: Recommendations of the Nomenclature Committee on Cell Death 2018. Cell Death Differ..

[19] De Carvalho Castro K.N., Lima D.F., Wolschick D., De Andrade I.M., Dos Santos  R.C., De Seixas dos Santos  F.J., Veras, L.M.C.; Costa-Júnior  L.M., Veras, L.M.C.; Costa-Júnior  L.M. (2016). In vitro effects of *Pilocarpus microphyllus* extracts and pilocarpine hydrochloride on *Rhipicephalus* (*Boophilus*) *microplus*. Rev. Bras. Parasitol. Veterinária.

[20] De Araujo Furtado M., Rossetti F., Chanda S., Yourick D. (2012). Exposure to nerve agents: From status epilepticus to neuroinflammation, brain damage, neurogenesis and epilepsy. Neurotoxicology.

[21] Yang W., Liao G., Hakim S.G., Ouyang D., Ringash J., Su Y. (2016). Is pilocarpine effective in preventing radiation-induced xerostomia? A systematic review and meta-analysis. Int. J. Radiat. Oncol. Biol. Phys..

[22] Saad E.A., Hassanien M.M., El-lban F.W. (2017). Nickel(II) diacetyl monoxime-2-pyridyl hydrazone complex can inhibit Ehrlich solid tumor growth in mice: A potential new antitumor drug. Biochem. Biophys. Res. Commun..

[23] Corso C.R., Acco A. (2018). Glutathione system in animal model of solid tumors: From regulation to therapeutic target. Crit. Rev. Oncol. Hematol..

[24] Mishra S., Tamta A.K., Sarikhani M., Desingu P.A., Kizkekra S.M., Pandit A.S., Kumar S., Khan D., Raghavan S.C., Sundaresan N.R. (2018). Subcutaneous Ehrlich Ascites Carcinoma mice model for studying cancer-induced cardiomyopathy. Sci. Rep..

[25] Weinberg R.A. (2008). The Biology of Cancer.

[26] Vendramini-Costa D.B., De Carvalho J.E. (2012). Molecular link mechanisms between inflammation and cancer. Curr. Pharm. Des..

[27] Dumitru C.A., Lang S., Brandau S. (2013). Modulation of neutrophil granulocytes in the tumor microenvironment: Mechanisms and consequences for tumor progression. Semin. Cancer Biol..

[28] Liang W., Ferrara N. (2016). The complex role of neutrophils in tumor angiogenesis and metastasis. Cancer Immunol. Res..

[29] Moses K., Brandau S. (2016). Human neutrophils: Their role in cancer and relation to myeloid-derived suppressor cells. Semin. Immunol..

[30] Houghton A.M. (2010). The paradox of tumor-associated neutrophils: Fueling tumor growth with cytotoxic substances. Cell Cycle.

[31] Winter C.A., Risley E.A., Nuss G.W. (1962). Carrageenin-induced edema in hind paw of the rat as an assay for antiinflammatory drugs. Exp. Biol. Med..

[32] Morris C.J. (2003). Carrageenan-induced paw edema in the rat and mouse. Methods in Molecular Biology (Clifton, N.J.).

[33] Vane J.R., Botting R.M. (1995). New insights into the mode of action of anti-inflammatory drugs. Inflamm. Res..

[34] Patil K.R., Mahajan U.B., Unger B.S., Goyal S.N., Belemkar S., Surana S.J., Ojha S., Patil C.R. (2019). Animal models of inflammation for screening of anti-inflammatory drugs: Implications for the discovery and development of phytopharmaceuticals. Int. J. Mol. Sci..

[35] Tubaro A., Dri P., Melato M., Mulas G., Bianchi P., Negro P., Della Loggia R. (1986). In the croton oil ear test the effects of non steroidal antiinflammatory drugs (NSAIDs) are dependent on the dose of the irritant. Agents Actions.

[36] Lapa A., Souccar C., Lima-Landman M., Castro M., Lima T. (2008). Métodos de Avaliação da Atividade Farmacológica de Plantas Medicinais.

[37] Fabri R.L., Garcia R.A., Florêncio J.R., De Castro Campos Pinto N., De Oliveira L.G., Aguiar J.A.K., Ribeiro A., Scio E. (2014). Anti-inflammatory and antioxidative effects of the methanolic extract of the aerial parts of *M itracarpus frigidus* in established animal models. J. Pharm. Pharmacol..

[38] Silva G.L., Luft C., Lunardelli A., Amaral R.H., Melo D.A., Donadio M.V., Nunes F.B., Azambuja M.S., Santana J.C., Moraes C. (2015). Antioxidant, analgesic and anti-inflammatory effects of lavender essential oil. An. Acad. Bras. Cienc..

[39] Carlson R.P., Lynn O.D., Chang J., Lewis A.J. (1985). Modulation of mouse ear edema by cyclooxygenase and lipoxygenase inhibitors and other pharmacologic agents. Agents Actions.

[40] Yang C.W., Chen W.L., Wu P.L., Tseng H.Y., Lee S.J. (2006). Anti-inflammatory mechanisms of phenanthroindolizidine alkaloids. Mol. Pharmacol..

[41] Chemler S.R. (2009). Phenanthroindolizidines and phenanthroquinolizidines: Promising alkaloids for anti-cancer therapy. Curr. Bioact. Compd..

[42] Hashmi M.A., Khan A., Farooq U., Khan S. (2018). Alkaloids as Cyclooxygenase Inhibitors in Anticancer Drug Discovery. Curr. Protein Pept. Sci..

[43] Queiroz R.F., Jordão A.K., Cunha A.C., Ferreira V.F., Brigagão M.R.P.L., Malvezzi A., Amaral A.T.D., Augusto O. (2012). Nitroxides attenuate carrageenan-induced inflammation in rat paws by reducing neutrophil infiltration and the resulting myeloperoxidase-mediated damage. Free Radic. Biol. Med..

[44] Bormio Nunes J.H., Bergamini F.R.G., Lustri W.R., Paiva P.P., Ruiz A.L.T.G., Carvalho J.E., Corbi P.P. (2017). Synthesis, characterization and in vitro biological assays of a silver(I) complex with 5-fluorouracil: A strategy to overcome multidrug resistant tumor cells. J. Fluor. Chem..

[45] Bormio Nunes J.H., Simoni D.A., Braga L.E.O., Ruiz A.L.T.G., Ernesto de Carvalho J., Corbi P.P. (2019). Synthesis, characterization, crystal structure and in vitro antiproliferative assays of the 2-thiouracilato(triphenylphosphine)gold(I) complex. J. Mol. Struct..

[46] National Cancer Institute Cell Lines in the In Vitro Screen. https://dtp.cancer.gov/discovery_development/nci-60/cell_list.htm.

[47] Franco Y.E.M., Okubo M.Y., Torre A.D., Paiva P.P., Rosa M.N., Silva V.A.O., Reis R.M., Ruiz A.L.T.G., Imamura P.M., De Carvalho J.E. (2019). Coronarin D induces apoptotic cell death and cell cycle arrest in human glioblastoma cell line. Molecules.

[48] Neto X.A.D.O., Alves A.C.S., Junior R.A.D., Rodrigues R.P., Lancellotti M., Almeida W.P., Kawano D.F. (2020). Molecular docking reveals the binding modes of anticancer alkylphospholipids and lysophosphatidylcholine within the catalytic domain of cytidine triphosphate: Phosphocholine cytidyltransferase. Eur. J. Lipid Sci. Technol..

[49] De Oliveira J.F., Da Silva A.L., Vendramini-Costa D.B., Da Cruz Amorim C.A., Campos J.F., Ribeiro A.G., De Moura R.O., Neves J.L., Ruiz A.L.T.G., De Carvalho J.E. (2015). Synthesis of thiophene-thiosemicarbazone derivatives and evaluation of their in vitro and in vivo antitumor activities. Eur. J. Med. Chem..

[50] Lin C.C., Yen M.H., Lo T.S., Lin C.F. (1997). The antiinflammatory and liver protective effects of Boehmeria nivea and B. nivea subsp. nippononivea in rats. Phytomedicine.

[51] Bradley P.P., Priebat D.A., Christensen R.D., Rothstein G. (1982). Measurement of Cutaneous Inflammation: Estimation of Neutrophil Content with an Enzyme Marker. J. Investig. Dermatol..

