# Supplementary material for: In Silico, In Vitro, and In Vivo Antitumor and Anti-Inflammatory Evaluation of a Standardized Alkaloid-Enriched Fraction Obtained from Boehmeria caudata Sw. Aerial Parts
Source: Molecules. 2020 Sep 3;25(17):4018. doi: 10.3390/molecules25174018 (PMC7504783; doi:10.3390/molecules25174018)
Supplement: Supplementary file 1 [file molecules-25-04018-s001.pdf]

Supplementary Material

In Silico, In Vitro, and In Vivo Antitumor and Anti-Inflammatory Evaluation of a Standardized Alkaloid-Enriched Fraction Obtained from *Boehmeria caudata* Sw. Aerial Parts

Paula P. de Paiva [1,2,3,*,†], Julia H. B. Nunes [2,4,†], Fabiana R. Nonato [2], Ana L. T. G. Ruiz [2,3], Rafael R. T. Zafred [1,2], Ilza M. O. Sousa [2,3], Márcia Y. Okubo [2,5], Daniel F. Kawano [3], Paula A. Monteiro [1,2], Mary A. Foglio [3] and João E. Carvalho [1,3]

[1] Institute of Biology, University of Campinas - UNICAMP, 13083-862, Campinas-SP, Brazil
[2] Chemical, Biological and Agricultural Pluridisciplinary Research Center (CPQBA), University of Campinas - UNICAMP, 13148-218 Paulínia-SP, Brazil.
[3] Faculty of Pharmaceutical Sciences, University of Campinas - UNICAMP, 13083-871 Campinas-SP, Brazil.
[4] Institute of Chemistry, University of Campinas - UNICAMP, 13083-970, Campinas-SP, Brazil.
[5] Piracicaba Dental School, University of Campinas, UNICAMP, 13414-903, Piracicaba-SP, Brazil.
* Correspondence: paula.p21@gmail.com; Tel +55-19-3521-7715
† These authors have contributed equally to the manuscript

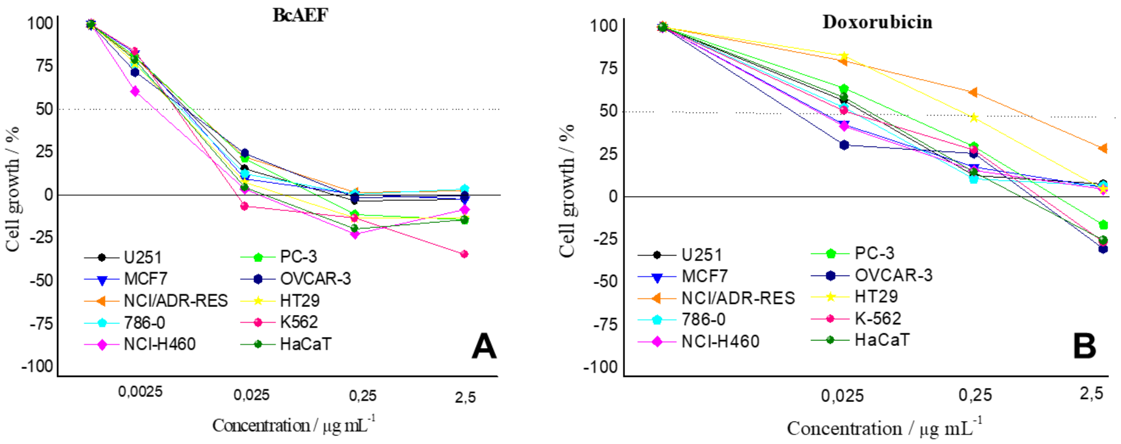

Figure S1. Cell growth profiles after 48 h exposure to (A) BcAEF (0.0025 to 2.5 μg mL^{-1}) and (B) doxorubicin (10^{-3} to 25 μg mL^{-1}). Human tumor cell lines: U251 (glioblastoma); MCF-7 (breast, adenocarcinoma); NCI-ADR/RES (multidrug resistant ovarian adenocarcinoma); 786-0 (kidney, adenocarcinoma); NCI-H460 (lung, large cells carcinoma); PC-3 (prostate, adenocarcinoma); OVCAR-3 (ovarian, adenocarcinoma); HT29 (colon, adenocarcinoma); K562 (chronic myeloid leukemia); Non-tumor human line: HaCat (immortal keratinocyte).

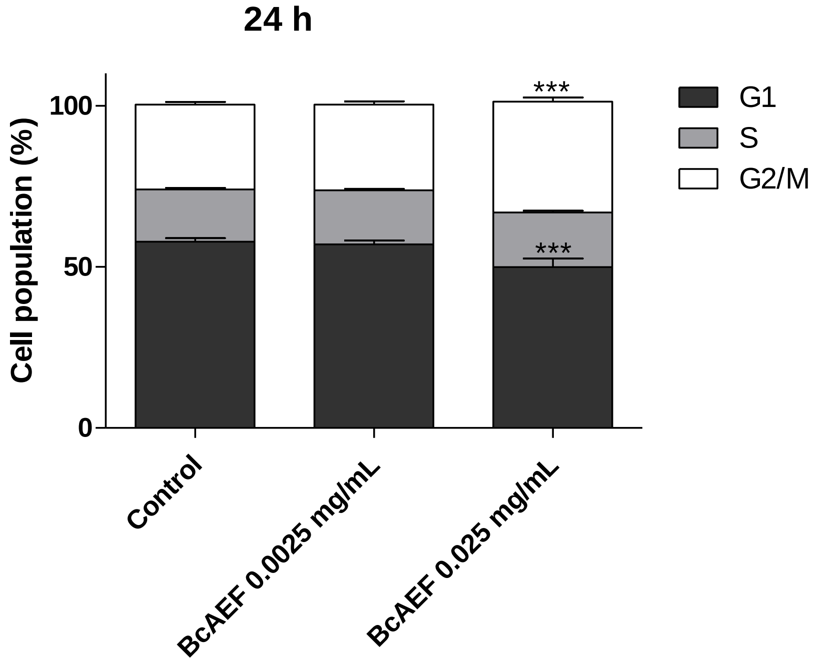

Figure S2. Influence of BcAEF on NCI-H460 cell cycle. NCI-H460 cells were treated for 24 h with 0.0025 and 0.025 μg/mL of BcAEF. Statistical analysis: 2 way-ANOVA followed by Bonferroni test (***p < 0.001, relative to untreated cells - control).

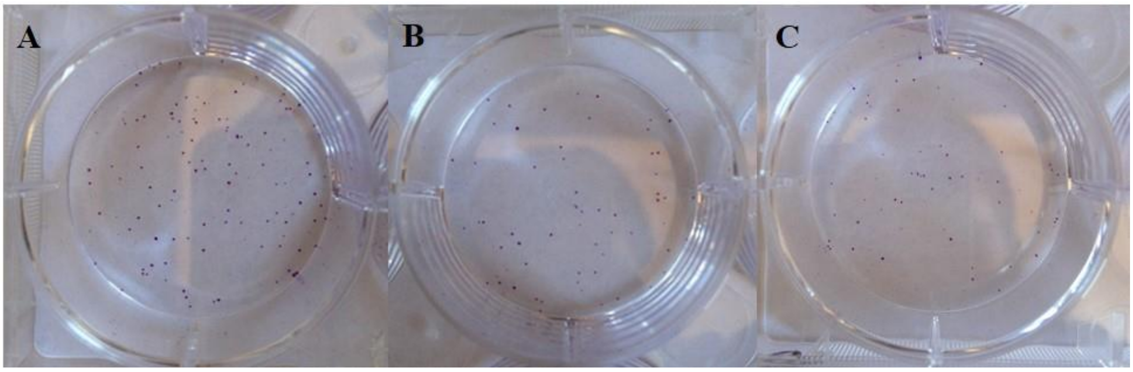

Figure S3. NCI-H460 colonies grown after 5 days, from (A) untreated cells (control), cells treated with (B) 0.0025 and (C) 0.025 μg mL^{-1} of BcAEF. Control: complete medium with 0.25% DMSO. Statistical analysis: One way ANOVA followed by Tukey test (***p < 0.001).

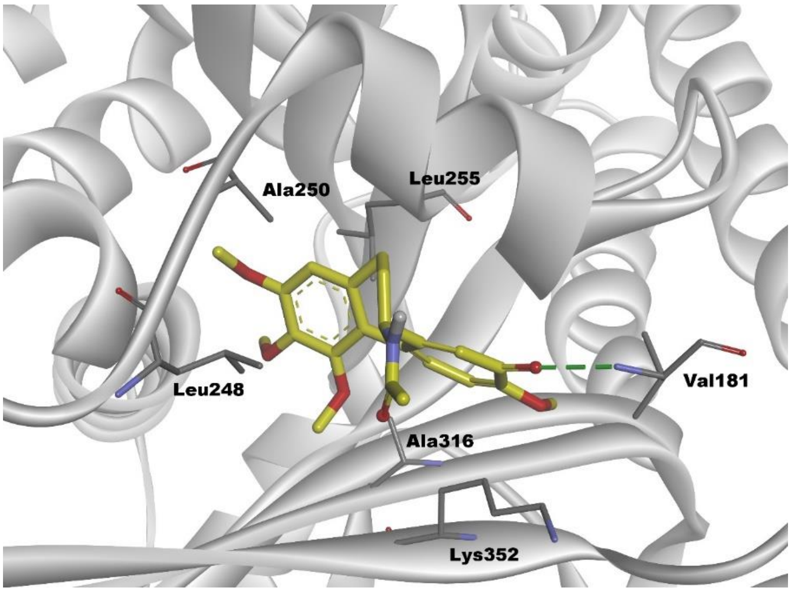

Figure S4. Docking model and main intermolecular interactions between colchicine and the amino acid residues from the colchicine biding site of *β*-tubulin. Carbonyl oxygen/Val^{181} (hydrogen bond; highlighted in dashed green lines; 3.05 Å), ring bond to carbonyl/Ala^{316} (CH/π; 5.45 Å), ring bond to carbonyl/Lys^{352} (CH/π; 4.20 Å), central ring/Leu^{248} (hydrophobic; 5.37 Å), central ring/Ala^{250} (hydrophobic; 4.86 Å), central ring/Leu^{255} (hydrophobic; 5.00 Å), trimethoxybenzene ring/Leu^{248} (CH/π; 3.78 Å), trimethoxybenzene ring/Ala^{250} (CH/π; 4.70 Å).

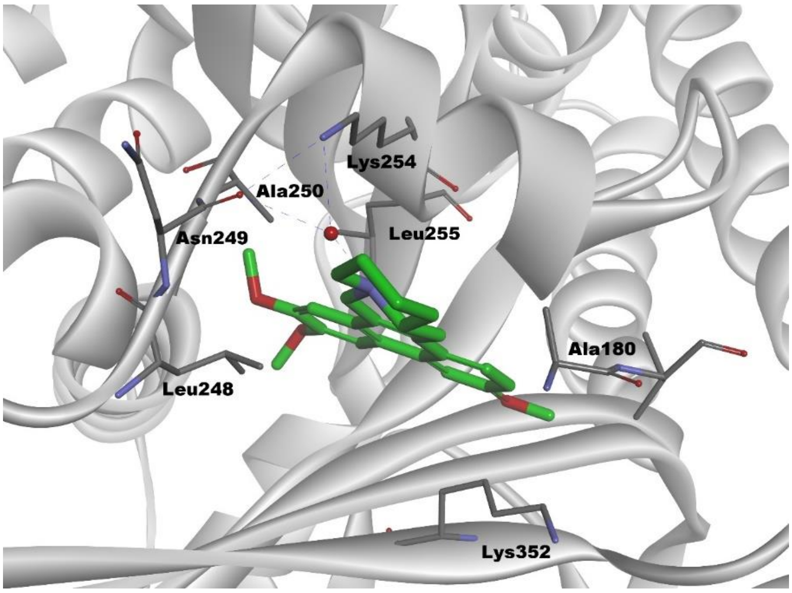

Figure S5. Docking model and main intermolecular interactions between *R*-boehmeriasin A and the amino acid residues from the colchicine biding site of *β*-tubulin. Quinolizidinic nitrogen/water/Asn^{249} (indirect hydrogen bond; highlighted as dashed blue lines; 3.08 Å, 2.92 Å), quinolizidinic nitrogen/water/Lys^{254} (indirect hydrogen bond; highlighted as dashed blue lines; 3.08 Å, 2.58 Å), peripheral quinolizidinic ring/Ala^{180} (hydrophobic; 4.80 Å), peripheral quinolizidinic ring/Ala^{250} (hydrophobic; 5.28 Å), peripheral quinolizidinic ring/Lys^{254} (hydrophobic; 5.33 Å), central quinolizidinic ring/Ala^{180} (hydrophobic; 4.61 Å), central quinolizidinic ring/Leu^{248} (hydrophobic; 5.12 Å), central quinolizidinic ring/Ala^{250} (hydrophobic; 4.80 Å), central aromatic ring/Ala^{250} (CH/π; 5.48 Å), central aromatic ring/Lys^{352} (CH/π; 5.26 Å), methoxybenzene ring/Lys^{352} (CH/π; 3.95 Å), methoxybenzene ring/Leu^{248} (CH/π; 3.82 Å), methoxybenzene ring/Ala^{250} (CH/π; 4.95 Å), methoxybenzene ring/Leu^{255} (CH/π; 4.59 Å).

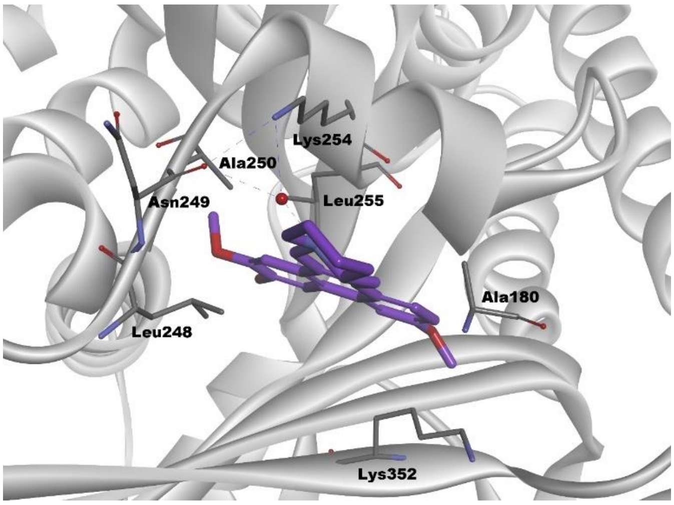

Figure S6. Docking model and main intermolecular interactions between *R*-boehmeriasin B and the amino acid residues from the colchicine biding site of *β*-tubulin. Quinolizidinic nitrogen/water/Asn^{249} (indirect hydrogen bond; highlighted as dashed blue lines; 3.02 Å, 2.92 Å), quinolizidinic nitrogen/water/Lys^{254} (indirect hydrogen bond; highlighted as dashed blue lines; 3.02 Å, 2.58 Å), peripheral quinolizidinic ring/Ala^{180} (hydrophobic; 4.81 Å), peripheral quinolizidinic ring/Ala^{250} (hydrophobic; 5.37 Å), peripheral quinolizidinic ring/Lys^{254} (hydrophobic; 5.45 Å), central quinolizidinic ring/Ala^{180} (hydrophobic; 4.34 Å), central quinolizidinic ring/Leu^{248} (hydrophobic; 5.23 Å), central quinolizidinic ring/Ala^{250} (hydrophobic; 5.38 Å), central aromatic ring/Ala^{250} (CH/π; 5.38 Å), central aromatic ring/Lys^{352} (CH/π; 5.39 Å), methoxybenzene ring/Lys^{352} (CH/π; 4.19 Å), hydroxymethoxybenzene ring/Leu^{248} (CH/π; 3.83 Å), hydroxymethoxybenzene ring/Ala^{250} (CH/π; 4.83 Å), hydroxymethoxybenzene ring/Leu^{255} (CH/π; 4.54 Å).

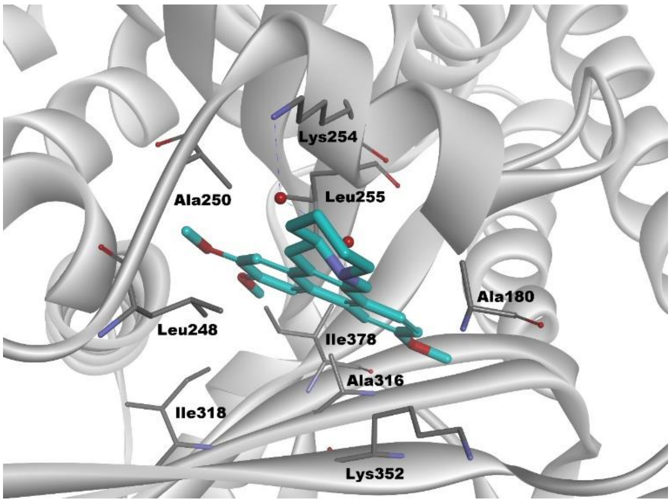

Figure S7. Docking model and main intermolecular interactions between *R*-cryptopleurine and the amino acid residues from the colchicine biding site of *β*-tubulin. Quinolizidinic nitrogen/ water/water/Lys^{254} (indirect hydrogen bond; highlighted as dashed blue lines; 1.71 Å, 2.83 Å, 2.58 Å), peripheral quinolizidinic ring/Ala^{180} (hydrophobic; 4.53 Å), peripheral quinolizidinic ring/Ala^{250} (hydrophobic; 5.14 Å), peripheral quinolizidinic ring/Lys^{254} (hydrophobic; 5.05 Å), central quinolizidinic ring/Ala^{180} (hydrophobic; 5.08 Å), central quinolizidinic ring/Leu^{248} (hydrophobic; 4.85 Å), central quinolizidinic ring/Ala^{250} (hydrophobic; 4.38 Å), central quinolizidinic ring/Lys^{254} (hydrophobic; 5.27 Å), central aromatic ring/Leu^{248} (CH/π; 5.34 Å), central aromatic ring/Ala^{250} (CH/π; 5.39 Å), central aromatic ring/Lys^{352} (CH/π; 5.43 Å), methoxybenzene ring/Ala^{316} (CH/π; 4.97 Å), methoxybenzene ring/Lys^{352} (CH/π; 4.03 Å), dimethoxybenzene ring/Leu^{248} (CH/π; 3.71 Å), dimethoxy-benzene ring/Ala^{250} (CH/π; 4.94 Å), dimethoxybenzene ring/Leu^{255} (CH/π; 3.58 Å), dimethoxybenzene ring/Leu^{248} methyl group (CH/π; 4.23 Å), dimethoxybenzene ring/Ala^{250} methyl group (CH/π; 3.68 Å), dimethoxybenzene ring/Ala^{316} methyl group (CH/π; 3.80 Å), dimethoxybenzene ring/Ile^{318} methyl group (CH/π; 5.23 Å), dimethoxybenzene ring/Ile^{378} methyl group (CH/π; 4.49 Å).

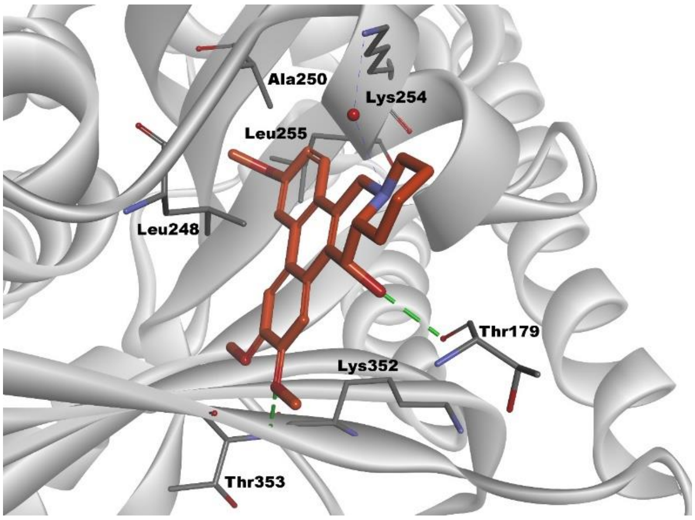

Figure S8. Docking model and main intermolecular interactions between *(-)-C* (15*R*)-hydroxycryptopleurine and the amino acid residues from the colchicine biding site of *β*-tubulin. Quinolizidinic nitrogen/water/Lys^{254} (indirect hydrogen bond; highlighted as dashed blue lines; 2.88 Å, 2.58 Å), hydroxyl group from quinolizidinic ring/Thr^{179} (hydrogen bond; highlighted in dashed green lines; 2.56 Å), central aromatic ring/Leu^{248} (CH/π; 3.14 Å, 3.14 Å, 3.52 Å), central aromatic ring/Ala^{250} (CH/π; 4.65 Å), methoxybenzene ring/Leu^{248} (CH/π; 3.92 Å), methoxybenzene/Ala^{250} (CH/π; 3.48 Å), methoxybenzene ring/Lys^{254} (CH/π; 5.24 Å), methoxybenzene/Leu^{255} (CH/π; 5.24 Å), dimethoxybenzene ring/Leu^{248} (CH/π; 3.61 Å), dimethoxybenzene ring/Lys^{352} (CH/π; 4.32 Å), methoxy group from dimethoxybenzene ring/Thr^{353} (hydrogen bond; highlighted in dashed green lines; 2.48 Å).

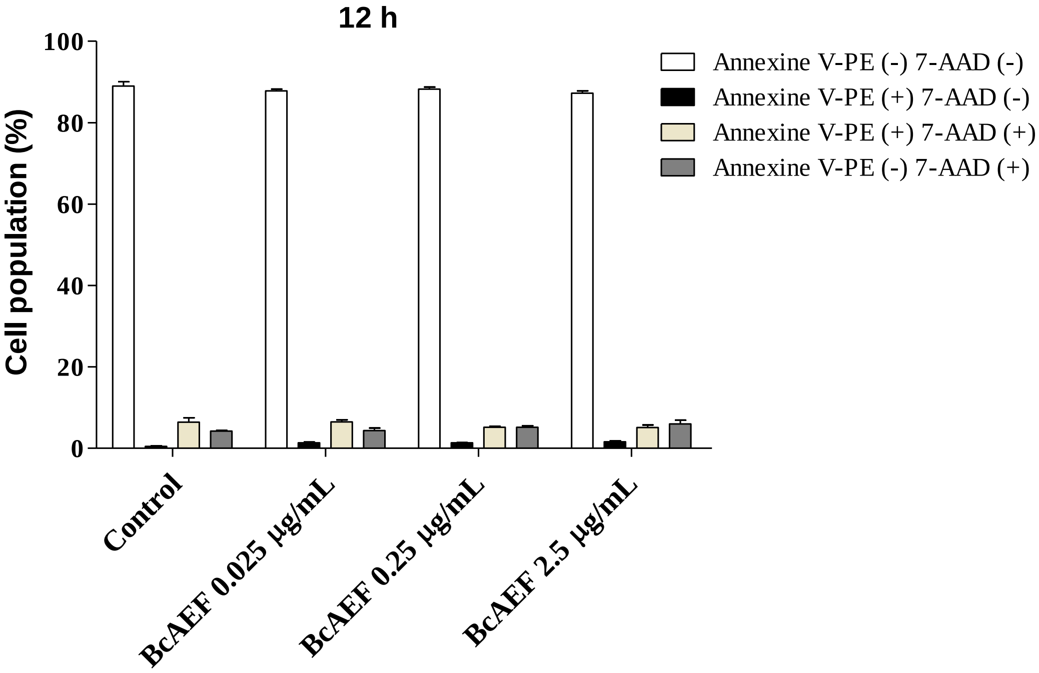

Figure S9. Influence of BcAEF on on phosphatidylserine externalization of NCI-H460 after 12 h of treatment. NCI-H460 cells were treated for 12 h with 0.025, 0.25 and 2.5 µg mL^{-1} of BcAEF. Statistical analysis: 2way-ANOVA followed by Bonferroni test (no statistical difference was found relative to untreated cells - control).

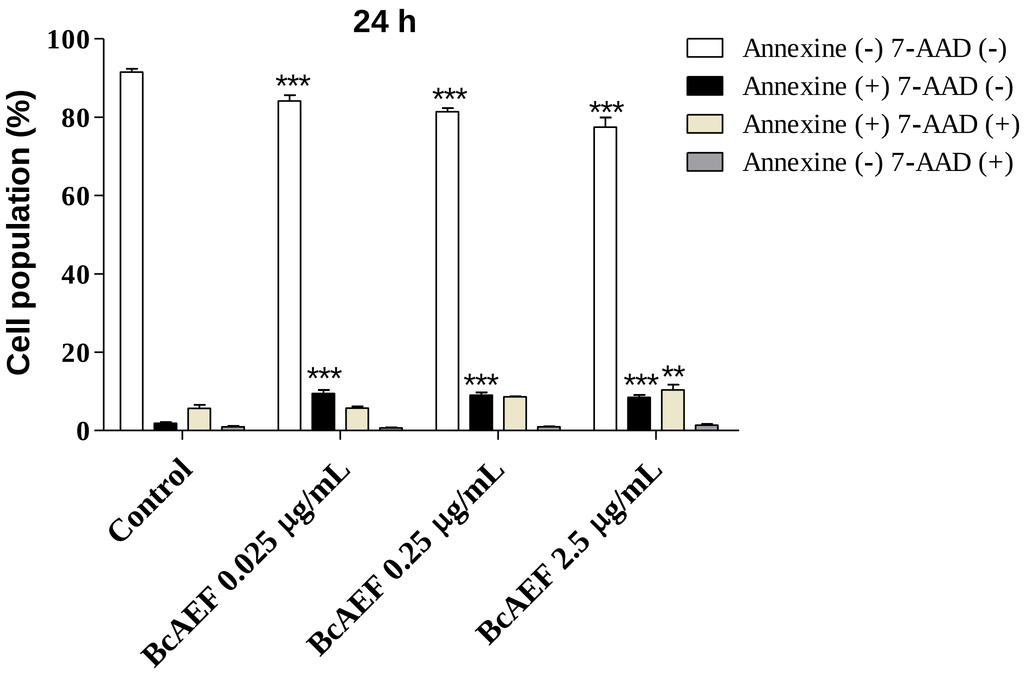

Figure S10. Influence of BcAEF on phosphatidylserine externalization of NCI-H460 after 24 h of treatment. NCI-H460 cells were treated for 24 h with 0.025, 0.25 and 2.5 µg mL^{-1} of BcAEF. Statistical analysis: 2way-ANOVA followed by Bonferroni test (**$p < 0.01$ and ***$p < 0.001$, relative to untreated cells - control).

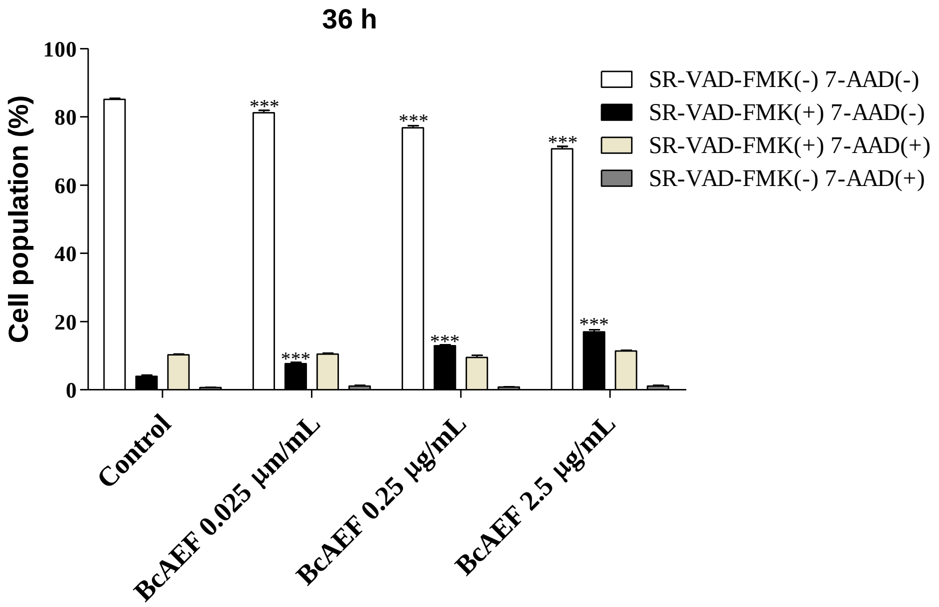

Figure S11. Influence of BcAEF on caspases activation of NCI-H460 after 36 h of treatment. NCI-H460 cells were treated for 36 h with 0.025, 0.25 and 2.5 µg mL^{-1} of BcAEF. Statistical analysis: 2way-ANOVA followed by Bonferroni test (***$p < 0.001$, relative to untreated cells - control).

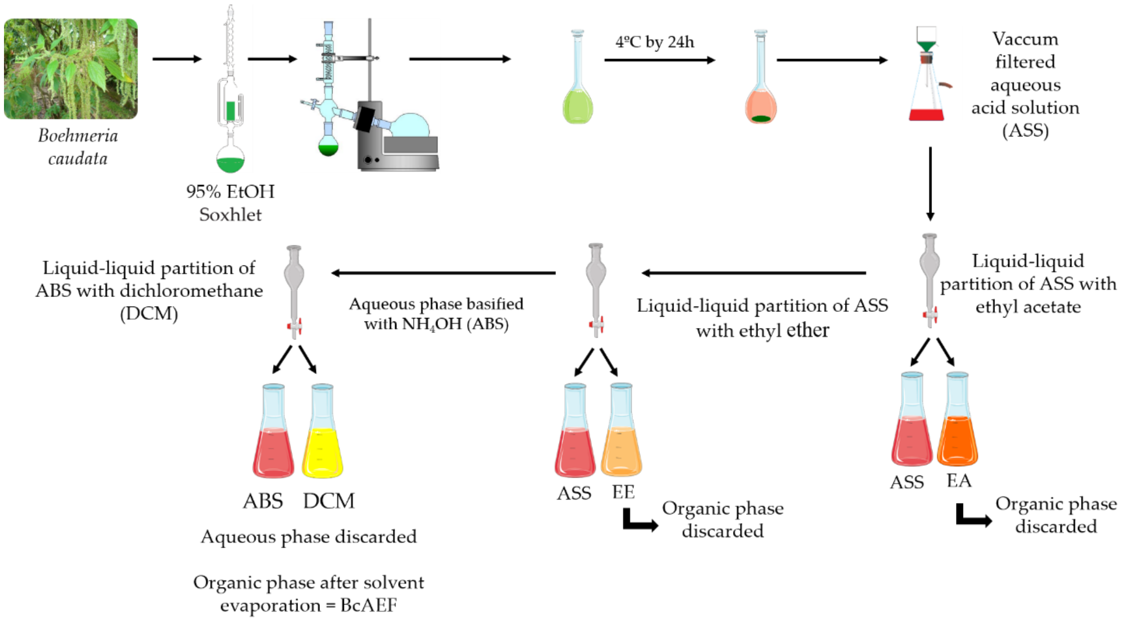

Figure S12. Scheme for BcAEF extraction from *Boehmeria caudata Sw.* aerial parts. From Kit Servier medical art-chemistry, 2020; Generalic, Eni "vacum filtration" and "soxhlet", 2020; Profpc "Rotavapor", 2020.

Table S1. Chemical structures and the respective docking scores calculated using GoldScore function (Gold 5.3 software) for colchicine, *R*-boehmeriasin A, and *R*-boehmeriasin B, *R*-cryptopleurine and (15*R*)-hydroxycryptopleurine.

Compound	Chemical structure	Docking score
Colchicine	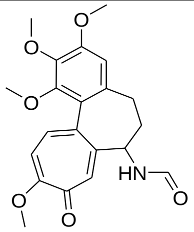	80.13
R-Boemeriasin A	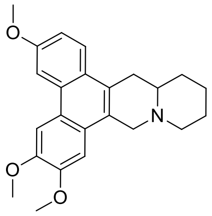	51.34
R-Boemeriasin B	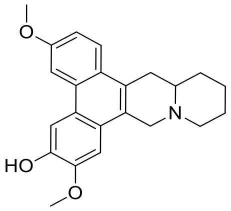	46.58

Table S1. *Cont.*

R-Cryptopleurine	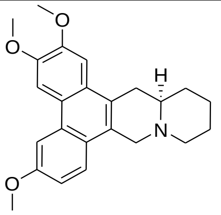	38.69
(-)-C (15*R*)-Hydroxycryptopleurine	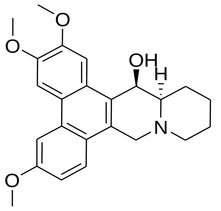	47.68

Table S2. Cell population distribution after using Annexin-V and 7-AAD dyes over NCI-H460 cells treated for 24 h and 36 h with BcAEF (0.025, 0.25 and 2.5 μg mL^{-1}). Control: untreated cells.

**BcAEF**	**Annexin(-) 7ADD(-)**	**Annexin(-) 7ADD(+)**	**Annexin(+) 7ADD(-)**	**Annexin(+) 7ADD(+)**
24 h, control	91.5 %	0.9 %	1.9 %	5.7 %
24 h, 0.025 μg mL^{-1}	84.1 % ***	0.6 % n.s.	9.5 % ***	5.7 % n.s.
24 h, 0.25 μg mL^{-1}	81.4 % ***	0.9 % n.s.	9.0 % ***	8.6 % n.s.
24 h, 2.5 μg mL^{-1}	77.5 % ***	1.4 % n.s.	8.5 % ***	10.4 % **
36 h, control	87.4 %	1.4 %	2.6 %	8.6 %
36 h, 0.025 μg mL^{-1}	85.6 % **	0.7 % n.s.	7.1 % ***	6.7 % **
36 h, 0.25 μg mL^{-1}	62.0 % ***	1.1 % n.s.	16.6 % ***	20.3 % ***
36 h, 2.5 μg mL^{-1}	54.3 % ***	2.0 % n.s.	17.9 % ***	25.7 % ***

**p < 0.01, ***p < 0.001; n.s. (not significantly different when compared to control), two-way ANOVA followed by Bonferroni test.

Table S3. Cell population distribution after using SR-VAD-FMK and 7-AAD dyes over NCI-H460 cells treated for 36 h and 48 h with BcAEF (0.025, 0.25 and 2.5 μg mL^{-1}). Control: untreated cells.

**BcAEF**	**SR-VAD-FMK(-) 7ADD(-)**	**SR-VAD-FMK(-) 7ADD(+)**	**SR-VAD-FMK(+) 7ADD(-)**	**SR-VAD-FMK(+) 7ADD(+)**
36h, control	85.1 %	0.6 %	4.0 %	10.3 %
36h, 0.025 μg mL^{-1}	81.2 % ***	1.1 % n.s.	7.6 % ***	10.4 % n.s.
36h, 0.25 μg mL^{-1}	76.8 % ***	0.8 % n.s.	12.9 %***	9.5 % n.s.
36h, 2.5 μg mL^{-1}	70.6 % ***	1.1 % n.s.	16.7 %***	11.4 % n.s.
48h, control	88.9 %	0.7 %	4.2 %	6.2 %
48h, 0.025 μg mL^{-1}	86.7 % ***	0.2 % n.s.	7.9 % ***	5.2 % n.s.
48h, 0.25 μg mL^{-1}	77.8 % ***	0.4 % n.s.	13.6 %***	8.2 % n.s.
48h, 2.5 μg mL^{-1}	70.0 % ***	1.5 % n.s.	18.5 %***	9.9 % ***

***$p < 0.001$; n.s. (not significantly different when compared to control), two-way ANOVA followed by Bonferroni test.

Table S4. Evaluation of edema variation and inhibition rate (%) promoted by BcAEF in the carrageenan-induced paw edema model.

		**1 hour**[a]		**2 hours**[a]		**4 hours**[a]		**6 hours**[a]	
**Group**	**Dose**	MV ± DP	%	MV ± DP	%	MV ± DP	%	MV ± DP	%
**Vehicle**	**10**[b]	25.39 ± 4.2	-	28.44 ± 5.6	-	25.30 ± 2.2	-	33.66 ± 4.3	-
**Dexa**	**25**[c]	27.67± 3.3	-8.97	24.51 ± 6.3	13.81	6.66 ± 6.5***	73.67	3.95 ± 6.5***	88.26
	**3** [c]	18.93 ± 2.1**	25.44	14.18 ± 5.5***	50.28	9.73 ± 4.3***	61.54	17.23 ± 5.4***	48.81
**BcAEF**	**10** [c]	14.14 ± 4.2***	44.36	9.96 ± 4.5***	64.97	3.19 ±2.2***	87.39	5.81 ± 7.3 ***	82.73
	**30** [c]	15.99 ±4.6 ***	37.02	10.87 ± 3.6***	61.77	4.77 ± 5.5***	81.14	6.69 ± 6.1***	80.72

a) Time after the edema induction, b) dose expressed in mL kg^{-1}, c) dose expressed in mg kg^{-1}. Edema variation: results are expressed as the mean ± standard deviation (MV ± SD); edema inhibition rate (%): difference of the edema variation of the treated groups in relation to the edema variation of the vehicle group divided by the edema variation of the vehicle group multiplied by one hundred. Groups: negative control (vehicle, PBS pH 7+ tween 80 5%, orally), positive control (dexa: dexamethasone 25 mg kg^{-1}, orally) and experimental groups (BcAEF 3, 10, and 30 mg kg^{-1}, orally), BcAEF: Alkaloid enriched fraction extracted from the aerial parts of *Boehmeria caudata* Sw. * p<0,05, ** p<0,01 and *** p<0,001, statistically significant difference in relation to vehicle group (Two way ANOVA – followed by Bonferroni test)

Table S5. Evaluation of reduction of edema, reduction rate of the neutrophil migration and the inhibition rate (%) promoted by BcAEF in the croton oil-induced ear edema model.

	Reduction oil-induced edema						Reduction rate of the neutrophil migration			
	Oral administration			Topic application			Oral administration		Topic application	
**Group**	**Dose**	**M[a]±SD**	**%**	**Dose**	**M[a]±SD**	**%**	**M[b]±SD**	**%**	**M[b]±SD**	**%**
**Vehicle**	10[c]	9.58 ± 2,11	-	20[d]	9.58 ± 2.11	-	0.480 ± 0.16[f]	-	0.249 ± 0.06[f]	-
**Dexa**	25c	1.48 ± 0.61***	84.55	5[e]	1.11 ± 0.581***	84.97	0.118 ± 0.07***	75.41	0.128 ± 0.05***	48.59
**BcAEF**	3[c]	1.8 ± 0.761***	81.21	3[e]	1.76 ± 1.271***	68.47	0.272 ± 0.13*[g]	43.55	0.156 ± 0.06[h]	37.34
	10[c]	3.12 ± 1.071***	67.43	10[e]	1.76 ± 0.841***	76.18	0.279 ± 0.09*[g]	41.87	0.115 ± 0.02***	53.81
	30[c]	2.82 ± 1.031***	70.56	30[e]	1.21 ± 0.861***	83.62	0.231 ± 0.05**[h]	51.87	0.120 ± 0.04***	51.80
**Naïve**	-	-	-	-	-	-	0.029 ± 0.01***	-	0.067 ± 0,01***	-

Results are expressed as the weights differences of equal portions obtained from the treated and untreated ears of the animals from each experimental group, been then calculated the averaging for the group (M ± SD: mean ± standard deviation). a) expressed in mg, b) expressed in optical density. Edema rate inhibition (%): difference of the edema variation of the treated groups in relation to the edema variation of the vehicle group divided by the edema variation of the vehicle group multiplied by one hundred. c) dose expressed in mg kg^{-1}. d) dose expressed in μL. e) dose expressed in mL kg^{-1}. Groups: negative control (vehicle, PBS pH 7+ tween 80 5%, orally), positive control (dexa: dexamethasone 25 mg kg^{-1}, orally and 5 mg mL^{-1} by topically) and experimental groups (BcAEF 3, 10, and 30 mg kg^{-1}, orally and topically), BcAEF: Alkaloid enriched fraction extracted from the aerial parts of *Boehmeria caudata* Sw. * p<0,05, ** p<0,01 and *** p<0,001, statistically significant difference in relation to vehicle group, significant difference by statistical means according to vehicle group. f: p<0.001, g: p<0.01, h: p<0.05, significant difference by statistical means according to satellite group (Two way ANOVA – followed by Bonferroni test).
